# Multiscale modelling the effects of CI genetic evolution in mosquito population on the control of dengue fever

**DOI:** 10.1038/s41598-017-13896-x

**Published:** 2017-10-24

**Authors:** Sha He, Xianghong Zhang, Juhua Liang, Sanyi Tang

**Affiliations:** School of Mathematics and Information Science, Shaanxi Normal University, Xi’an, 710119 P.R. China

## Abstract

Endosymbiotic *Wolbachia* bacteria are widely applied for the control of dengue fever by manipulating the reproductive mechanism of mosquitoes, including maternal inheritance and cytoplasmic incompatibility (CI). CI means that the offsprings from the matings between *Wolbachia* infected males and uninfected females can not be hatched. At present, CI effect is assumed as a constant in most of dynamic systems for the spread of *Wolbachia*. However, their spread may arouse the evolution of mosquitoes to resist CI. Thus, a multiscale model combining a birth-pulse model with a gene-induced discrete model for the frequencies of alleles is proposed to describe the spread of *Wolbachia* in mosquito population with resistance allele of CI. The main results indicate that the strategy of population eradication can not be realized, while the strategy of population replacement may be realized with the success of sensitive or resistance allele. If appropriate *Wolbachia* strains can not be selected, then there is a high probability of the failure of population replacement. Moreover, *Wolbachia*-induced parameters may arouse the catastrophic shifts among stable states of the model. In addition, the demographic parameters and *Wolbachia*-induced parameters may affect the level and the speed of population replacement and the density of uninfected mosquitoes.

## Introduction

Dengue fever as one type of vector-borne diseases, is caused by dengue virus mainly transmitted by mosquitoes^1,2^. This disease seriously threaten human health, especially in tropical and subtropical regions due to geographical distribution of mosquitoes, climatic characteristics and human movement patterns^3,4^. At present, endosymbiotic *Wolbachia* bacteria has been discovered to control dengue diseases as a new promising biological approach. *Wolbachia* is preferable to females and can be maternally transmitted from female mosquitoes to their offsprings^5,6^. *Wolbachia* exhibits a special phenomenon during the mating of their hosts, named as cytoplasmic incompatibility (CI)^7,8^. CI can be expressed as the death of the mosquito embryos when uninfected females mate with *Wolbachia* infected males whereas other types of matings are not affected^9,10^.

Owing to maternal inheritance and CI mechanism, *Wolbachia* can only vertically spread from females to their offsprings and terminate in infected males, which benefits to enhancing the fitness of infected hosts and reducing the proportion of uninfected hosts^11,12^. So when *Wolbachia* is utilized in the control of vector-borne diseases, we hope to realize two strategies, one is population suppression (eradication) and other is population replacement (*Wolbachia* infected insects established and replacing uninfected ones). Since *Wolbachia* bacteria is widely applied for the control of dengue fever, various mathematical models^13–17^ have been established to explain the dynamics of *Wolbachia* invasion and the change on the size of host population. Especially, Zhang *et al*.^18,19^ developed impulsive models to investigate the effects of different *Wolbachia* infected mosquito augmentations, including releases with different sex and release quantity, release moment and period, release sequence and times, on the success of different strategies of mosquito control.

In general, the spread of *Wolbachia* is not complete and often has a cost to offset host fitness because of intragenomic conflict and coevolution^20–23^. Martłnez-Rodrłguez *et al*.^20^ proposed a matrix model to describe the variation of infection proportion in the host life cycle and predicted the evolution progress, and showed that the long-term infection was affected by the change of *Wolbachia* proportion. Turelli^21^ proposed that the host genome should evolve to ameliorate action of *Wolbachia*, further he analyzed selection on *Wolbachia* and their host genes by infection frequency models. Cooper *et al*.^24^ discovered that *Wolbachia* can cause intraspecific and interspecific CI based on the three hybridizing species of *Drosophila*, and both host genotype and *Wolbachia* variation modulate CI expression.

In addition, the dynamics of CI-expressing *Wolbachia* infections of insects have been extensively modeled^25–27^. Assuming random mating and discrete generations on host population, Caspari *et al*.^25,26^ proposed difference equations to describe the frequency of infected adults at generation *t* + 1. They proved that there exist infected adult eradicated and established equilibria, and for *Wolbachia* spreading into the host population it needs to be introduced at an initial prevalence greater than the value of unstable equilibrium. In *D*. *melanogaster*, male development time can greatly influence CI expression discovered by Yamada *et al*.^28^. Thus, the infected males taking longer to undergo larval development quickly lose their ability to express the CI phenotype. Not only that, the level of CI expression in *Drosophila melanogaster* declines rapidly with male age, particularly when males are repeatedly mated^29^. Besides, Hurst *et al*.^30^ improved the recursions to describe the dynamics of CI, and pointed that following the invasion of CI inducing *Wolbachia* into an uninfected population, not only will the sterilizing effect wane but the conditions become permissive for the spread of the uninfected cytotype.

Based on above works, it is interesting to investigate questions about the evolution of infections and their hosts, combining with their breeding process. However, a population-genetic model described the spread of insecticide resistance on mosquito population had been developed by Birget *et al*.^31^. In fact, the development of mosquito resistance to insecticide and resistance of CI are similar, we aim to explore the effect of resistance development of mosquito populations to CI mechanism on the spread of *Wolbachia* among mosquito populations by mathematical method. To do this, we assume that the evolution of CI mechanism is determined by a single gene with two alleles which conforms to Hardy-Weinberg Principle^32^. Thus the frequencies of alleles in infected and uninfected mosquitoes will change with the reproductive process of mosquito populations. Note that four factors may affect the reproductive progress, including maternal inheritance, resistance level, the dominance level of gene and the fertility cost. Although the frequencies of alleles are independent to the density of host population, while the latter may be impacted by the former. Therefore, we combine a birth-pulse model^33^ of mosquito populations with a discrete model of the frequencies of alleles, i.e. the multiscale models have been developed in the present work.

## Models and Methods

### Birth-pulse model

In order to study the effect of the evolution of *Wolbachia*-induced CI on the dynamic behaviors of mosquito populations, we first introduce the following birth-pulse model^33–35^

2.1 $$\{\begin{array}{l}\begin{array}{l}\tfrac{dI(t)}{dt}=-(\delta +D)I(t)N(t),\\ \tfrac{dU(t)}{dt}=-\delta U(t)N(t),\end{array}\}\,t\ne nT,\\ \begin{array}{l}I(n{T}^{+})=(1+\tau b(N(t)))I(nT),\\ U(n{T}^{+})=U(nT)+b(N(t))\,\mathrm{((1}-\tau )I(nT)+U(nT)\,(1-q\tfrac{I(nT)}{N(nT)}))\end{array}\}\,t=nT,\end{array}$$

where *n*(*n* = 1, 2, …) denotes the sequence of birth pulse and *T* is the breading period. *N*(*t*) is the total density of mosquito population, which includes the infected *I*(*t*) and uninfected *U*(*t*). The densities of infected and uninfected mosquitoes are decreased owing to density-dependent death whenever *t* ≠ *nT*, while birth pulses with the CI mechanism and matrilineal inheritance occur at *t* = *nT*. The items *I*(*nT* ^+^) and *U*(*nT* ^+^) denote the mosquito densities of the infected and uninfected ones at time *nT* after birth pulse. The description of parameters are shown in Table 1. For more details of above model, please refer to the work in^33^.

**Table 1:** Parameter descriptions, values and sources for the models.

Para.	Description	Value (Range)	Source
*b*	Mosquito natural birth rate	[1.5, 3]	39
*δ*	Mosquito natural death rate	[0.01, 0.05]	39
*D*	Extra death rate of *Wol*. infected mosquitoes	(−0.033, 0.1518)	19
*T*	Mosquito population reproductive period	{3, 5, 10}	19
*τ*	Maternal transmission rate of *Wol*. infected females	[0, 1]	Assumed
*z*	The fitness cost of fertility for resistant homozygote	[0, 1]	Assumed
*ρ*	Resistance level to CI	[0, 1]	Assumed
*h*	The dominance level of resistance conferred by CI resistance allele	[0, 1]	Assumed
*p*_*I*,*n*_ (*p*_*U*,*n*_)	Frequency of allele *R* in *Wol*. infected (uninfected) population at generation *n*	[0, 1]	
*q*_*I*,*n*_ (*q*_*U*,*n*_)	Frequency of allele *S* in *Wol*. infected (uninfected) population at generation *n*	[0, 1]	
*P*_1,*RR*_ (*P*_1,*RS*_, *P*_1,*SS*_)	Frequency of genotype *RR*(*RS*, *SS*) for *Wol*. infected females mating with all the males at generation *n*	[0, 1]	
*P*_2,*RR*_ (*P*_2,*RS*_, *P*_2,*SS*_)	Frequency of genotype *RR*(*RS*, *SS*) for *Wol*. uninfected females mating with uninfected males at generation *n*	[0, 1]	
*P*_3,*RR*_ (*P*_3,*RS*_, *P*_3,*SS*_)	Frequency of genotype *RR*(*RS*, *SS*) for *Wol*. uninfected females mating with infected males at generation *n*	[0, 1]	
*W*_1,*RR*_ (*W*_1,*RS*_, *W*_1,*SS*_)	Fitness of genotype *RR*(*RS*, *SS*) for CI nonoccurrence	\	
*W*_2,*RR*_ (*W*_2,*RS*_, *W*_2,*SS*_)	Fitness of genotype *RR*(*RS*, *SS*) for CI occurrence	\	
*Q*_*I*,*RR*_ (*Q*_*I*,*RS*_, *Q*_*I*,*SS*_)	Frequency of genotype *RR*(*RS*, *SS*) for new born *Wol*. infected offsprings at generation *n* + 1	[0, 1]	
*Q*_*U*,*RR*_ (*Q*_*U*,*RS*_, *Q*_*U*,*SS*_)	Frequency of genotype *RR*(*RS*, *SS*) for new born *Wol*. uninfected offsprings at generation *n* + 1	[0, 1]	

*Wol*.: *Wolbachia*.

### Birth-pulse model with CI genetic evolution

Suppose that the spread of *Wolbachia* and the evolution of CI in mosquito population are determined by a single gene with two alleles, including dominant (here resistance) allele *R* and recessive (here sensitive) allele *S*. It gives rise to three different genotypes in mosquito population, including resistant homozygote (*RR*), heterozygote (*RS*) and sensitive homozygote (*SS*).

#### The frequencies of three genotypes

The genotype frequencies for both males and females are assumed to be equal. The frequencies of alleles *R* and *S* in *Wolbachia* infected (uninfected) mosquitoes at generation *n* are denoted as *p*_*I*,*n*_ and *q*_*I*,*n*_ (*p*_*U*,*n*_ and *q*_*U*,*n*_), respectively, then we have *p*_*I*_ + *q*_*I*_ = *I*/(*I* + *U*) and *p*_*U*_ + *q*_*U*_ = *U*/(*I* + *U*). Thus, if the density of mosquitoes is zero, then *p*_*I*,*n*_ + *q*_*I*,*n*_ + *p*_*U*,*n*_ + *q*_*U*,*n*_ = 0, otherwise *p*_*I*,*n*_ + *q*_*I*,*n*_ + *p*_*U*,*n*_ + *q*_*U*,*n*_ = 1. According to Hardy-Weinberg Principle and maternal inheritance, the frequencies of alleles satisfy the equation ((*p*_*I*,*n*_ + *p*_*U*,*n*_) + (*q*_*I*,*n*_ + *q*_*U*,*n*_))^2^ = 1. The fitnesses of three genotypes are different at generation *n* owing to CI effect in the progress of genetic evolution, which causes the frequencies of alleles in infected and uninfected mosquitoes change constantly. Thus, it is necessary to investigate the frequency dynamics of alleles. The frequencies of different genotypes with different mating types *P*_*i*_(*i* = 1, 2, 3) at generation *n* are shown in Table 2.

**Table 2:** The frequencies of three genotypes with different mating types.

Genotype	*P*_1_ : *I* × (*I or U*)	*P*_2_ : *U* × *U*	*P*_3_ : *U* × *I*
RR	${p}_{I,n}^{2}+{p}_{I,n}{p}_{U,n}$	${p}_{U,n}^{2}$	*p*_*U*,*n*_ *p*_*I*,*n*_
RS	2*p*_*I*,*n*_ *q*_*I*,*n*_ + *p*_*I*,*n*_ *q*_*U*,*n*_ + *q*_*I*,*n*_ *p*_*U*,*n*_	2*p*_*U*,*n*_ *q*_*U*,*n*_	*p*_*U*,*n*_ *q*_*I*,*n*_ + *q*_*U*,*n*_ *p*_*I*,*n*_
SS	${q}_{I,n}^{2}+{q}_{I,n}{q}_{U,n}$	${q}_{U,n}^{2}$	*q*_*U*,*n*_ *q*_*I*,*n*_

×: Mate.

#### The fitnesses of three genotypes

**Figure 1:**
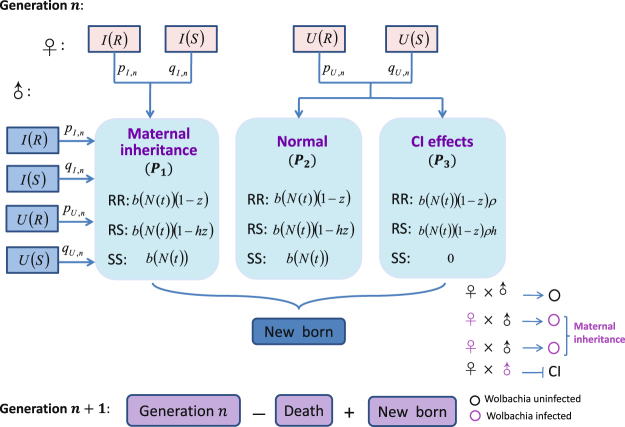
Flow diagram of the fitnesses from different mating types of *Wolbachia* infected and uninfected mosquitoes at generation *n*. Total density of mosquito population is divided into four classes, including infected females, infected males, uninfected females and uninfected males, with three genotypes *RR*, *RS* and *SS*. Assume that there is the same sex ratio in infected and uninfected mosquitoes. The fitnesses *W*_*i*_(*i* = 1, 2) from three mating types *P*_1_, *P*_2_ and *P*_3_ with three genotypes are shown in Table 2. When infected females mate with males (*P*_1_), their offsprings may be infected owing to maternal inheritance; when uninfected females mate with uninfected males (*P*_2_), their offsprings are uninfected; while when uninfected females mate with infected males (*P*_3_), their offsprings may not be hatched owing to CI mechanism, so the three mating types *P*_1_, *P*_2_ and *P*_3_ represent maternal inheritance, normal and CI effect groups, respectively. The densities of mosquitoes at generation *n* + 1 are composed of the survivals of mosquitoes at generation *n* and new born offsprings from generation *n*.

Note that *Wolbachia* infected females never undergo CI mechanism, and all the female mosquitoes mating with uninfected males can not be affected by CI. For convenience, these matings without CI mechanism are renamed as group one. However, uninfected females mating with infected males may arise the occurrence of CI which is renamed as group two. The reproduction of mosquito, which is determined by the natural birth rate *b*(*N*(*t*)) and their resistance of CI effect, called fitness^31^. In the group one, the effect of resistant homozygote individuals *RR* on the fertility cost of resistance is assumed as *z*, then the cost in heterozygote individuals *RS* is the product of *z* and the level of dominance *h*, homozygote sensitives is not affected by the resistance and reproduce normally. For the group two, we assume that all the sensitive mosquitoes *SS* can not reproduce and not be affected by the resistance, that is complete CI mechanism will occur. There is a survival of resistant homozygote *RR* because of the resistance *ρ* and the cost of fertility is reduced by *z*. The fitness in heterozygote individuals *RS* is the product of *ρ* and the level of dominant allele *h*, meanwhile, the cost of fertility is also reduced by *hz*. Therefore, the fitnesses of different genotypes in different groups at generation *n* are calculated as shown in Table 3, with all the parameter values of *ρ*, *h* and *z* belonging to  $[0,1]$. The detailed flow diagram of genetic evolution is shown in Fig. 1.

**Table 3:** The fitnesses of three genotypes in different groups.

Genotype	Group one (*W*_1_)	Group two (*W*_2_)
RR	*W*_1,*RR*_ = *b*(*N*(*t*)) (1 − *z*)	*W*_2,*RR*_ = *b*(*N*(*t*)) (1 − *z*)*ρ*
RS	*W*_1,*RS*_ = *b*(*N*(*t*)) (1 − *hz*)	*W*_2,*RS*_ = *b*(*N*(*t*)) (1 − *hz*)*ρh*
SS	*W*_1,*SS*_ = *b*(*N*(*t*))	*W*_2,*SS*_ = 0

#### The genotype frequencies of new born offsprings

The genotype frequencies of new born offsprings at generation *n* + 1 are determined by the frequencies and the fitnesses of three genotypes *RR*, *RS* and *SS*. Infected females occur maternal inheritance with a probability *τ* (also called as transmission rate *τ* for convenience), or in other words, infected females can produce a proportion 1 − *τ* of uninfected offsprings. So the ratios of new born infected and uninfected mosquitoes from three different genotypes to the total mosquito population at generation *n* are shown in Table 4.

**Table 4:** The genotype frequencies of new born *Wolbachia* infected and uninfected offsprings.

Genotype	*Wolbachia* infected (*I*)	*Wolbachia* uninfected (*U*)
RR	${Q}_{I,RR}=\tfrac{{P}_{\mathrm{1,}RR,n}{W}_{\mathrm{1,}RR}\tau }{1+Q}$	${Q}_{U,RR}=\tfrac{{P}_{\mathrm{1,}RR,n}{W}_{\mathrm{1,}RR}\mathrm{(1}-\tau )+{P}_{\mathrm{2,}RR,n}{W}_{\mathrm{1,}RR}+{P}_{\mathrm{3,}RR,n}{W}_{\mathrm{2,}RR}}{1+Q}$
RS	${Q}_{I,RS}=\tfrac{{P}_{\mathrm{1,}RS,n}{W}_{\mathrm{1,}RS}\tau }{1+Q}$	${Q}_{U,RS}=\tfrac{{P}_{\mathrm{1,}RS,n}{W}_{\mathrm{1,}RR}\mathrm{(1}-\tau )+{P}_{\mathrm{2,}RS,n}{W}_{\mathrm{1,}RR}+{P}_{\mathrm{3,}RS,n}{W}_{\mathrm{2,}RR}}{1+Q}$
SS	${Q}_{I,SS}=\tfrac{{P}_{\mathrm{1,}SS,n}{W}_{\mathrm{1,}SS}\tau }{1+Q}$	${Q}_{U,SS}=\tfrac{{P}_{\mathrm{1,}SS,n}{W}_{\mathrm{1,}RR}\mathrm{(1}-\tau )+{P}_{\mathrm{2,}SS,n}{W}_{\mathrm{1,}RR}+{P}_{\mathrm{3,}SS,n}{W}_{\mathrm{2,}RR}}{1+Q}$

With *Q* = (*P*_1,*RR*,*n*_ *W*_1,*RR*_ + *P*_1,*RS*,*n*_ *W*_1,*RS*_ + *P*_1,*SS*,*n*_ *W*_1,*SS*_) + (*P*_2,*RR*,*n*_ *W*_1,*RR*_ + *P*_2,*RS*,*n*_ *W*_1,*RS*_ + *P*_2,*SS*,*n*_ *W*_1,*SS*_) + (*P*_3,*R)R*,*n*_ *W*_2,*RR*_ + *P*_3,*RS*,*n*_ *W*_2,*RS*_ + *P*_3,*SS*,*n*_ *W*_2,*SS*_).

#### The frequencies of two alleles

**Figure 2:**
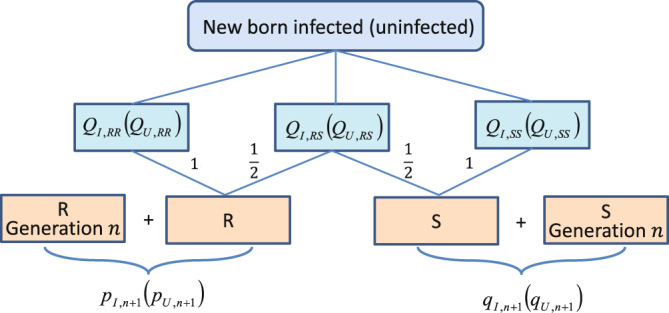
The frequencies of alleles in *Wolbachia* infected and uninfected mosquitoes at generation *n* + 1. The frequencies consist of the three genotype frequencies of new born part and the survivals from generation *n*.

Since the development of mosquito populations is a process of overlapping generation, the survival mosquitoes from generation *n* may affect the frequencies of alleles *R* and *S* in infected and uninfected mosquitoes at generation *n* + 1. So the frequencies of alleles at generation *n* + 1 consist of the survival mosquitoes from generation *n* and the new born offsprings at generation *n* + 1. Therefore, the process of the frequencies of alleles in infected and uninfected mosquitoes from generation *n* to *n* + 1 is depicted by Fig. 2 and the following equations

22 $$\{\begin{array}{rcl}{p}_{I,n+1} & = & \frac{{p}_{I,n+1}}{Q+1}+{Q}_{I,RR}+0.5{Q}_{I,RS},\\ {q}_{I,n+1} & = & \frac{{q}_{I,n+1}}{Q+1}+{Q}_{I,SS}+0.5{Q}_{I,RS},\\ {p}_{U,n+1} & = & \frac{{p}_{U,n+1}}{Q+1}+{Q}_{U,RR}+0.5{Q}_{U,RS},\\ {q}_{U,n+1} & = & \frac{{q}_{U,n+1}}{Q+1}+{Q}_{U,SS}+0.5{Q}_{U,RS},\end{array}$$

where *Q* is the total birth rate of *Wolbachia* infected and uninfected mosquitoes at generation *n* with genotypes *RR*, *RS* and *SS*, while *Q*_*I*,*RR*_, *Q*_*I*,*RS*_ and *Q*_*I*,*SS*_ (*Q*_*U*,*RR*_, *Q*_*U*,*RS*_ and *Q*_*U*,*SS*_) denote the frequencies of genotypes *RR*, *RS* and *SS* for new born *Wolbachia* infected (uninfected) offsprings at generation *n* + 1, respectively. Based on (2.1), (2.2) and Table 2, a multiscale model combining the birth-pulse model with the genetic evolution to resist CI mechanism in mosquito populations is proposed as follows

2.3 $$\{\begin{array}{l}\begin{array}{l}\,\tfrac{dI(t)}{dt}=-(\delta +D)I(t)N(t),\\ \tfrac{dU(t)}{dt}=-\delta U(t)N(t),\end{array}\}\,t\ne nT,\\ \begin{array}{l}\begin{array}{l}\,I(n{T}^{+})=I(nT)+({Q}_{I,RR}+{Q}_{I,RS}+{Q}_{I,SS})\,(Q+\mathrm{1)}N(nT),\\ U(n{T}^{+})=U(nT)+({Q}_{U,RR}+{Q}_{U,RS}+{Q}_{U,SS})\,(Q+\mathrm{1)}N(nT),\end{array}\\ \begin{array}{l}{p}_{I,n+1}=\frac{{p}_{I,n}}{Q+1}+{Q}_{I,RR}+0.5{Q}_{I,RS},\\ \,{q}_{I,n+1}=\frac{{q}_{I,n}}{Q+1}+{Q}_{I,SS}+0.5{Q}_{I,RS},\\ {p}_{U,n+1}=\frac{{p}_{U,n}}{Q+1}+{Q}_{U,RR}+0.5{Q}_{U,RS},\\ \,{q}_{U,n+1}=\frac{{q}_{U,n}}{Q+1}+{Q}_{U,SS}+0.5{Q}_{U,RS},\end{array}\end{array}\}\,t=nT,\end{array}$$

where one scale is based on the dynamic system of mosquito populations, and another is based on the frequencies of alleles in mosquito populations. The parameter interpretations and notations in the model are given in Table 1.

### Stroboscopic map of system (2.3)

The stroboscopic map which plays an important role in investigating the dynamic behavior of system (2.3) is determined by the densities of infected and uninfected mosquitoes after each birth pulse at discrete time *nT*. Note that we aim to study the effect of CI genetic evolution on the density of mosquito population. For simplicity, birth rates of both infected and uninfected mosquitoes are assumed to be equal and as a constant *b*. In addition, we mainly study the case when extra death rate of infected mosquitoes *D* = 0, which only affects the change rates for the densities of mosquito populations, but does not change the qualitative behavior of system (2.3)^17^.

To obtain stroboscopic map of system (2.3), we first solve the two differential equations of system (2.3) in any birth pulse interval (*nT*, (*n* + 1)*T*] as follows

2.4 $$\{\begin{array}{l}\,I(t)=\frac{I(n{T}^{+})}{\delta (t-nT)\,(I(n{T}^{+})+U(n{T}^{+}))+1},\\ U(t)=\frac{U(n{T}^{+})}{\delta (t-nT)\,(I(n{T}^{+})+U(n{T}^{+}))+1}\mathrm{.}\end{array}$$

Denote *I*_*n*_ = *I*(*nT* ^+^), *U*_*n*_ = *U*(*nT* ^+^), and deform some equations, we obtain the corresponding stroboscopic map of system (2.3) as follows

25 $$\{\begin{array}{l}\begin{array}{l}{I}_{n+1}=\tfrac{{I}_{n}}{\delta T({I}_{n}+{U}_{n})+1}+\tfrac{{I}_{n}+{U}_{n}}{\delta T({I}_{n}+{U}_{n})+1}\tau {Q}_{1}\triangleq {f}_{1}({I}_{n},{U}_{n},{p}_{I,n},{q}_{I,n},{p}_{U,n},{q}_{U,n}),\\ {U}_{n+1}=\tfrac{{U}_{n}}{\delta T({I}_{n}+{U}_{n})+1}+\tfrac{{I}_{n}+{U}_{n}}{\delta T({I}_{n}+{U}_{n})+1}[\mathrm{(1}-\tau ){Q}_{1}+{Q}_{2}]\triangleq {f}_{2}({I}_{n},{U}_{n},{p}_{I,n},{q}_{I,n},{p}_{U,n},{q}_{U,n}),\end{array}\}\,({\rm{I}})\\ \begin{array}{l}{p}_{I,n+1}=\tfrac{{p}_{I,n}+\tau A}{Q+1}\triangleq {f}_{3}({p}_{I,n},{q}_{I,n},{p}_{U,n},{q}_{U,n}),\\ {q}_{I,n+1}=\tfrac{{q}_{I,n}+\tau B}{Q+1}\triangleq {f}_{4}({p}_{I,n},{q}_{I,n},{p}_{U,n},{q}_{U,n}),\\ {p}_{U,n+1}=\tfrac{{p}_{U,n}+\mathrm{(1}-\tau )A+C}{Q+1}\triangleq {f}_{5}({p}_{I,n},{q}_{I,n},{p}_{U,n},{q}_{U,n}),\\ {q}_{U,n+1}=\tfrac{{q}_{U,n}+\mathrm{(1}-\tau )B+F}{Q+1}\triangleq {f}_{6}({p}_{I,n},{q}_{I,n},{p}_{U,n},{q}_{U,n}),\end{array}\}\,({\rm{II}})\end{array}$$

where *Q* = *Q*_1_ + *Q*_2_ with *Q*_1_ = *A* + *B*, *Q*_2_ = *C* + *F*,

$$\begin{array}{l}A=b(({p}_{I,n}^{2}+{p}_{I,n}{p}_{U,n})\,\mathrm{(1}-z)+({p}_{I,n}{q}_{I,n}+\tfrac{1}{2}{p}_{I,n}{q}_{U,n}+\tfrac{1}{2}{p}_{U,n}{q}_{I,n})\,\mathrm{(1}-hz)),\\ B=b(({p}_{I,n}{q}_{I,n}+\tfrac{1}{2}{p}_{I,n}{q}_{U,n}+\tfrac{1}{2}{p}_{U,n}{q}_{I,n})\,\mathrm{(1}-hz)+({q}_{I,n}^{2}+{q}_{I,n}{q}_{U,n})),\\ C=b(({p}_{U,n}^{2}+\rho {p}_{U,n}{p}_{I,n})\,\mathrm{(1}-z)+({p}_{U,n}{q}_{U,n}+\tfrac{1}{2}\rho h{p}_{U,n}{q}_{I,n}+\tfrac{1}{2}\rho h{p}_{I,n}{q}_{U,n})\,\mathrm{(1}-hz)),\\ F=b(({p}_{U,n}{q}_{U,n}+\tfrac{1}{2}\rho h{p}_{U,n}{q}_{I,n}+\tfrac{1}{2}\rho h{p}_{I,n}{q}_{U,n})\,\mathrm{(1}-hz)+{q}_{U,n}^{2})\mathrm{.}\end{array}$$

Based on system (2.5), the densities of infected and uninfected mosquitoes depend on variables *p*_*i*_ and *q*_*i*_ (*i* = *I*, *U*), while the frequencies of alleles *R* and *S* in infected and uninfected mosquitoes are independent to the densities of infected and uninfected mosquitoes. Therefore, system (2.5) is divided into two subsystems, and the first two equations are renamed as subsystem (I) and the other four equations as subsystem (II). For convenience, denote the right hand functions of subsystem (I) as *f*_*i*_ (*i* = 1, 2), while the right hand functions of subsystem (II) as *f*_*i*_ (*i* = 3, 4, 5, 6). Since subsystem (II) is independent to subsystem (I), we first analyze it in the coming section.

## Results

### The analysis of subsystem (II)

The existence and stabilities of equilibria for subsystem (II) in different cases are shown in Table 5, while for their detailed calculation and analysis, see in Supplementary Information (SI). The expressions of equilibria for subsystem (II) are listed as follows

3.6 $$\{\begin{array}{l}{E}_{0}^{\ast }=\mathrm{(0},0,0,\mathrm{0)},{E}_{1}^{\ast }=\mathrm{(0},0,{p}_{U},1-{p}_{U}),\\ {E}_{1}^{\mathrm{(1)}}=\mathrm{(0},0,1,\mathrm{0)},{E}_{1}^{\mathrm{(2)}}=\mathrm{(0},0,0,1).\\ {E}_{2}^{\ast }=(0,\frac{1+\sqrt{{{\rm{\Delta }}}_{1}}}{2},\,0,\,\frac{1-\sqrt{{{\rm{\Delta }}}_{1}}}{2}),{E}_{3}^{\ast }=(\mathrm{0,}\,\frac{1-\sqrt{{{\rm{\Delta }}}_{1}}}{2},\,\mathrm{0,}\,\frac{1+\sqrt{{{\rm{\Delta }}}_{1}}}{2}),\\ {E}_{4}^{\ast }=(\frac{\mathrm{(1}-\rho )+\sqrt{{{\rm{\Delta }}}_{2}}}{\mathrm{2(1}-\rho )},\,\mathrm{0,}\,\frac{\mathrm{(1}-\rho )-\sqrt{{{\rm{\Delta }}}_{2}}}{\mathrm{2(1}-\rho )},\,0),\\ {E}_{5}^{\ast }=(\frac{\mathrm{(1}-\rho )-\sqrt{{{\rm{\Delta }}}_{2}}}{\mathrm{2(1}-\rho )},\,\mathrm{0,}\,\frac{\mathrm{(1}-\rho )+\sqrt{{{\rm{\Delta }}}_{2}}}{\mathrm{2(1}-\rho )},\,0),\\ {E}_{6}^{\ast }=({p}_{I},\,1-{p}_{I},\,\mathrm{0,}\,\mathrm{0).}\end{array}$$

In the following, we mainly analyze subsystems (I) and (II) without fertility cost, and the effects of fertility cost and dominance level on the speed of population replacement are considered in subsection 3.5. In order to understand the existence of equilibria of subsystem (II) in more detail, we take *τ* and *ρ* as bifurcation parameters, and four key lines are defined as follows

$${L}_{1}\,:\,\rho =1,\,{L}_{2}\,:\,\tau =0.75,\,{L}_{3}\,:\,\rho =4\tau -3,\,{L}_{4}\,:\,\tau =1.$$

**Figure 3:**
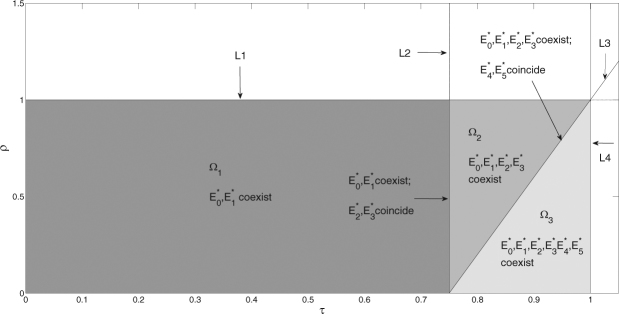
Two parameter bifurcation for the existence of equilibria of subsystem (II). *L*_1_ : *ρ* = 1, *L*_2_ : *τ* = 0.75, *L*_3_ : *ρ* = 4*τ* − 3, *L*_4_ : *τ* = 1.

Then the four lines (*L*_*i*_, *i* = 1, 2, 3, 4) divide the *τ* and *ρ* parameter space into eight regions as shown in Fig. 3. Equilibria ${E}_{i}^{\ast }(i=0,\,\mathrm{1)}$, ${E}_{i}^{\ast }(i=0,\,1,\,2,\,\mathrm{3)}$ and ${E}_{i}^{\ast }(i=0,\,1,\,2,\,\ldots ,\,\mathrm{5)}$ coexist in regions Ω_1_, Ω_2_ and Ω_3_, respectively. Especially, equilibria ${E}_{i}^{\ast }(i=2,\,\mathrm{3)}$ and ${E}_{i}^{\ast }(i=4,\,\mathrm{5)}$ coincide in lines *L*_2_ and *L*_3_, respectively.

**Table 5:** The existence and stabilities of equilibria for subsystem (II) in different cases.

Equilibrium	${\bar{{\boldsymbol{E}}}}_{{\bf{0}}}^{{\boldsymbol{\ast }}}$	${\bar{{\boldsymbol{E}}}}_{{\bf{1}}}^{{\boldsymbol{\ast }}}$		${\bar{{\boldsymbol{E}}}}_{{\bf{2}}}^{{\boldsymbol{\ast }}}$	${\bar{{\boldsymbol{E}}}}_{{\bf{3}}}^{{\boldsymbol{\ast }}}$	${\bar{{\boldsymbol{E}}}}_{{\bf{4}}}^{{\boldsymbol{\ast }}}$	${\bar{{\boldsymbol{E}}}}_{{\bf{5}}}^{{\boldsymbol{\ast }}}$
*τ* ∈ (0, 1), *z* = 0	S	S		S	U	S	U
*τ* = 1, *z* = 0	S	S		${E}_{6}^{\ast }$			
				S			
*τ* ∈ (0, 1), *z* ∈ (0, 1]	S	${E}_{1}^{\mathrm{(1)}}$	${E}_{1}^{\mathrm{(2)}}$	S	U	S	U
		U	S				
*τ* = 1, *z* ∈ (0, 1]	S	${E}_{1}^{\mathrm{(1)}}$	${E}_{1}^{\mathrm{(2)}}$	\			
		U	S				

S: Stable, U: Unstable, \: nonexistence.

Next we state the biology significance for the five stable equilibria ${E}_{0}^{\ast }$, ${E}_{1}^{\ast }$, ${E}_{2}^{\ast }$, ${E}_{4}^{\ast }$ and ${E}_{6}^{\ast }$. Only in the neighbourhood of ${E}_{0}^{\ast }$, the solutions of subsystem (II) tend to ${E}_{0}^{\ast }$, so it is difficult to make the frequencies of alleles in infected and uninfected mosquitoes tend to zero at the same time, that is to say, it is difficult to eradicate infected and uninfected mosquitoes simultaneously. Thus in the following, we focus on the other four equilibria. When the solutions of system (II) stabilize at equilibrium ${E}_{1}^{\ast }$, then all alleles to depict CI effect are in uninfected mosquitoes, which indicates that some uninfected mosquitoes have resistance allele, while the rest of uninfected mosquitoes only have recessive allele. The stability of equilibrium ${E}_{2}^{\ast }$ indicates that the success of recessive allele in mosquito population, and it means that all mosquitoes don’t have resistance allele. However when the solutions of system (II) stabilize at ${E}_{4}^{\ast }$, contrary to ${E}_{2}^{\ast }$, then the resistance allele can succeed, such that all mosquito population has resistance allele. The stability of equilibrium ${E}_{6}^{\ast }$, contrary to ${E}_{1}^{\ast }$, indicates that all alleles to depict CI effect are all in infected mosquitoes, which indicates that some infected mosquitoes have resistance allele, while the rest of infected mosquitoes only have recessive allele.

### Basins of attraction of subsystem (II)

Based on the above subsection, the stabilities of equilibria of subsystem (II) have been determined by analytical and numerical techniques. In order to study the effect of different initial frequencies of alleles *R* and *S* in infected and uninfected mosquitoes on the final distribution of the allele frequencies, it is necessary to further study the effects of different initial values on the solutions of subsystem (II).

**Figure 4:**
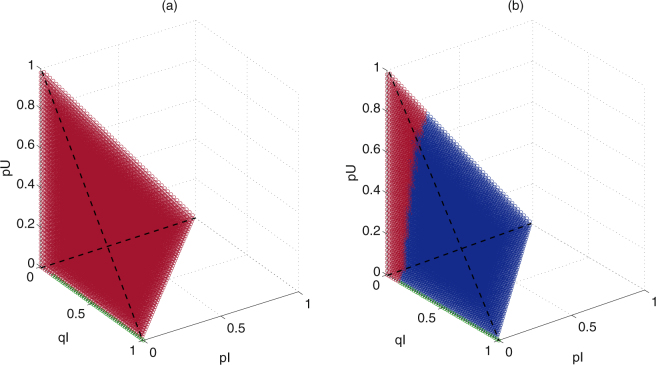
Basins of attraction of subsystem (II) with different parameter regions. All the initial values are from region Ω = {*p*_*I*_ ≥ 0, *q*_*I*_ ≥ 0, *p*_*U*_ ≥ 0, *q*_*U*_ ≥ 0, *p*_*I*_ + *q*_*I*_ + *p*_*U*_ + *q*_*U*_ ≤ 1}. (**a**) Basins of attraction of equilibria ${E}_{1}^{\ast }$ and ${E}_{2}^{\ast }$ with *τ* and *ρ* lying in Ω_2_ (see Fig. 3) and *ρ* = 0.8. (**b**) Basins of attraction of equilibria ${E}_{1}^{\ast }$, ${E}_{2}^{\ast }$ and ${E}_{4}^{\ast }$ with *τ* and *ρ* lying in Ω_3_ (see Fig. 3) and *ρ* = 0.5. When initial frequencies of subsystem (II) are chosen from the deep red, green and blue regions, then its solutions stabilize at equilibria ${E}_{1}^{\ast }$, ${E}_{2}^{\ast }$ and ${E}_{4}^{\ast }$, respectively. Parameter values are fixed as follows: *b* = 2, *δ* = 0.03, *T* = 5, *τ* = 0.9, *ρ* = 0.5, *h* = 0.35, *z* = 0.

To do this, all parameter values are fixed, except *ρ*, and a wide ranges of frequencies of alleles *R* and *S* in infected and uninfected mosquitoes are chosen, then we obtain the basins of attraction of equilibria for subsystem (II), as shown in Fig. 4. When parameter values are chosen from region Ω_2_, then it follows from Figs 3 and 4(a) that the two stable equilibria ${E}_{1}^{\ast }$ and ${E}_{2}^{\ast }$ coexist. Consequently the solutions of subsystem (II) from the initial frequencies of alleles lying in deep red or green region will stabilize at equilibrium ${E}_{1}^{\ast }$ or ${E}_{2}^{\ast }$, respectively, which indicates that the two regions are the basins of attraction of the two equilibria, respectively. Especially, in this case, although there exist initial frequencies of alleles in infected mosquitoes, most solutions of subsystem (II) will tend to equilibrium ${E}_{1}^{\ast }$ such that the frequencies of alleles tending to zero in infected mosquitoes has a high probability 99.81%, which is obtained by calculating the ratio of the number of points in deep red region to that of in region Ω. All those confirm that a high probability for the failure of population replacement.

Similarly, Figs 3 and 4(b) show that the tri-stable equilibria ${E}_{1}^{\ast }$, ${E}_{2}^{\ast }$ and ${E}_{4}^{\ast }$ coexist when parameter values are chosen from region Ω_3_, then the solutions of subsystem (II) from the initial frequencies of alleles lying in deep red, green or blue region will stabilize at equilibrium ${E}_{1}^{\ast }$, ${E}_{2}^{\ast }$ or ${E}_{4}^{\ast }$, respectively, which indicates that the three regions are the basins of attraction of the three equilibria, respectively. In this case, most of solutions of subsystem (II) will tend to equilibrium ${E}_{1}^{\ast }$ or ${E}_{4}^{\ast }$ such that there is a small probability (9.87%) for the frequencies of alleles tending zero in infected mosquitoes or a significant probability (89.94%) for the success of resistance allele in mosquito populations. All those confirm that we will fail to realize population replacement but can realize partial population replacement with resistance allele.

Figure 4(a,b) show that there is a low probability for the solutions of subsystem (II) stabilizing at equilibrium ${E}_{2}^{\ast }$, that is to say, the probability for the establishment of populations with sensitive allele owing to their low fitness to CI is low.

### Parameter bifurcation analyses of subsystem (II)

**Figure 5:**
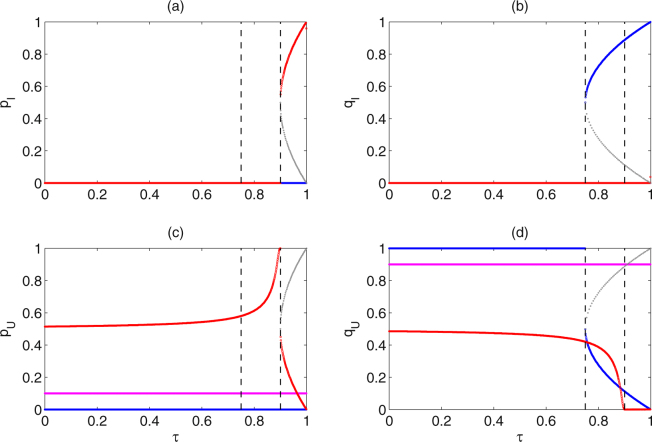
Catastrophic bifurcations of the frequencies of alleles at equilibria of subsystem (II) from three different initial frequencies of alleles versus *τ*. The gray curves denote the corresponding bifurcation of unstable equilibria ${E}_{3}^{\ast }$ and ${E}_{5}^{\ast }$. The magenta, blue and red curves denote the bifurcations of initial frequencies of alleles (*p*_*I*,1_, *q*_*I*,1_, *p*_*U*,1_, *q*_*U*,1_) at (0, 0, 0.1, 0.9), (0, 0.8, 0, 0.2) and (0.1, 0.1, 0.4, 0.4), respectively. Baseline parameter values are fixed as follows: *b* = 2, *δ* = 0.03, *T* = 5, *ρ* = 0.6, *h* = 0.35, *z* = 0.

**Figure 6:**
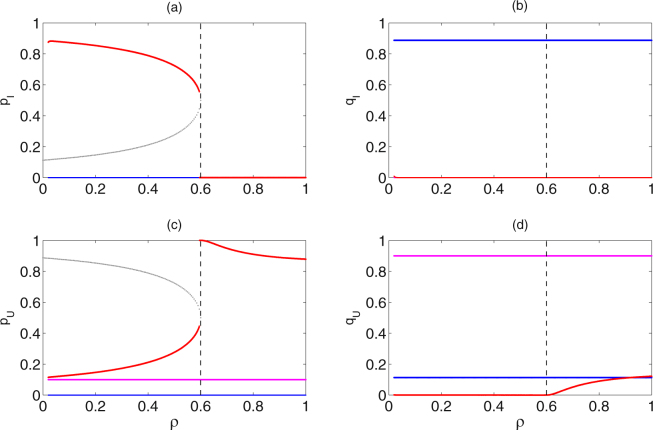
Catastrophic bifurcations of the frequencies of alleles at equilibria of subsystem (II) from three different initial frequencies of alleles versus *ρ*. Initial frequencies of alleles and Baseline parameter values are the same as those in Fig. 5, except for *τ* = 0.9.

**Figure 7:**
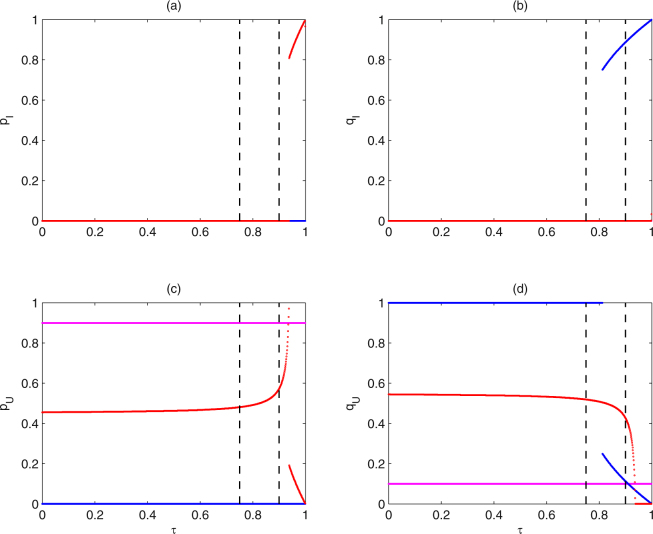
The switching of stable equilibria of subsystem (II) from different initial frequencies of alleles versus *τ*. The magenta, blue and red curves denote the corresponding switches for subsystem (II) from initial frequencies of alleles (*p*_*I*,1_, *q*_*I*,1_, *p*_*U*,1_, *q*_*U*,1_) as (0, 0, 0.9, 0.1), (0, 0.25, 0, 0.75) and (0.05, 0.05, 0.4, 0.5), respectively. Baseline parameter values are the same as those in Fig. 5.

**Figure 8:**
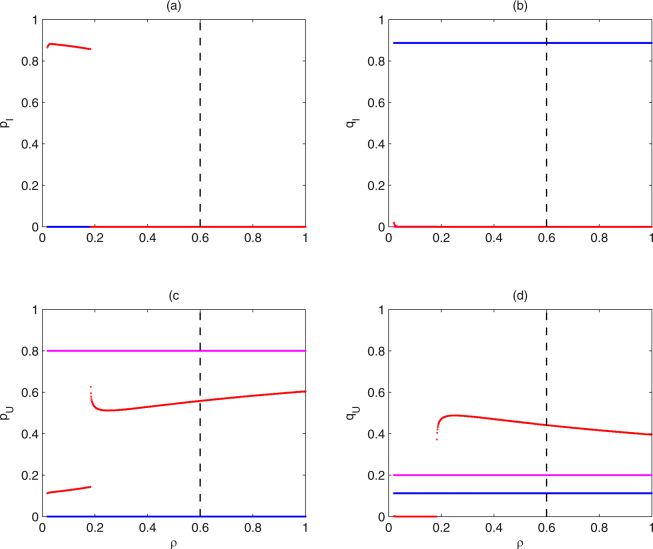
The switching of stable equilibria of subsystem (II) from different initial frequencies of alleles versus *ρ*. The magenta, blue and red curves denote the corresponding switches for subsystem (II) from initial frequencies of alleles (*p*_*I*,1_, *q*_*I*,1_, *p*_*U*,1_, *q*_*U*,1_) as (0, 0, 0.8, 0.2), (0, 0.5, 0, 0.5) and (0, 0.12, 0.33, 0.55), respectively. Baseline parameter values are the same as those in Fig. 5.

According to Fig. 3, the existence of stable equilibrium ${E}_{1}^{\ast }$, the coexistence of two stable equilibria ${E}_{1}^{\ast }$ and ${E}_{2}^{\ast }$, and the coexistence of three stable equilibria ${E}_{1}^{\ast }$, ${E}_{2}^{\ast }$ and ${E}_{4}^{\ast }$ are in regions Ω_1_, Ω_2_ and Ω_3_, respectively. In order to investigate the distribution of allele frequencies, it is important to study the effects of different parameters and initial frequencies on the coexistence, catastrophic shifts and the values of three stable equilibria. Note that for the special case *z* = 0, the level of dominance *h* does not appear in the expressions of equilibria, so these equilibria are mainly determined by parameters *τ* and *ρ*. Therefore, we separately fix the two parameters, and plot one or two-parameter bifurcation diagrams from three different initial values, as shown in Figs 5, 6, 7 and 8.

From Fig. 5, when the transmission rate is low (*τ* ∈ (0, 3/4]), then the frequencies of alleles in infected mosquitoes tend to zero for all three initial values. With the increase of transmission rate (*τ* ∈ (3/4, (3 + *ρ*)/4]), it occurs the coexistence of two attractors ${E}_{1}^{\ast }$ and ${E}_{2}^{\ast }$. As the transmission rate further increase (*τ* ∈ ((3 + *ρ*)/4, 1]), the three attractors ${E}_{1}^{\ast }$, ${E}_{2}^{\ast }$ and ${E}_{4}^{\ast }$ can coexist. Especially, with the increase of transmission rate *τ*, the solution of subsystem (II) from initial frequencies of alleles (*p*_*I*,1_, *q*_*I*,1_, *p*_*U*,1_, *q*_*U*,1_) = (0, 0, 0.1, 0.9) can stabilize at ${E}_{1}^{\ast }$, and the catastrophic shift of equilibria does not occur at all (see magenta curves); the stable states of subsystem (II) from initial frequencies of alleles (*p*_*I*,1_, *q*_*I*,1_, *p*_*U*,1_, *q*_*U*,1_) = (0, 0.8, 0, 0.2) switch from ${E}_{1}^{\ast }$ to ${E}_{2}^{\ast }$ at *τ* = 3/4 (see blue curves); the stable states of subsystem (II) from initial frequencies of alleles (*p*_*I*,1_, *q*_*I*,1_, *p*_*U*,1_, *q*_*U*,1_) = (0.1, 0.1, 0.4, 0.4) switch from ${E}_{1}^{\ast }$ to ${E}_{4}^{\ast }$ at *τ* = (*ρ* + 3)/4 (see red curves).

Similarly, it follows from Fig. 6 that when the resistance level is low (*ρ* ∈ (0, 4*τ* − 3]), then it occurs the existence of three attractors ${E}_{1}^{\ast }$, ${E}_{2}^{\ast }$ and ${E}_{4}^{\ast }$. With the increase of resistance level (*ρ* ∈ (4*τ* − 3, 1]), it occurs the coexistence of two attractors ${E}_{1}^{\ast }$ and ${E}_{2}^{\ast }$. Especially, with the increase of resistance level *ρ*, the solutions of subsystem (II) from the same initial frequencies of alleles as those of the deep magenta and blue curves in Fig. 5 stabilize at ${E}_{1}^{\ast }$ and ${E}_{2}^{\ast }$, respectively, and there is no switch of equilibria (see magenta and blue curves); the stable states of subsystem (II) from the same initial frequencies of alleles as those of the red curves switch from ${E}_{4}^{\ast }$ to ${E}_{1}^{\ast }$ at *ρ* = 4*τ* − 3 (see red curves).

In the following, it is necessary to further study how initial frequencies of alleles affect the catastrophic shift and the values of stable equilibria of subsystem (II), as shown in Figs 7 and 8. It follows from Fig. 7, with the increase of transmission rate *τ*, the solutions of subsystem (II) from initial frequencies of alleles (*p*_*I*,1_, *q*_*I*,1_, *p*_*U*,1_, *q*_*U*,1_) = (0, 0, 0.9, 0.1) stabilize at ${E}_{1}^{\ast }$, and the catastrophic shift of equilibria does not occur at all (see magenta curves); the stable states of subsystem (II) from initial frequencies of alleles (*p*_*I*,1_, *q*_*I*,1_, *p*_*U*,1_, *q*_*U*,1_) = (0, 0.25, 0, 0.75) switch from ${E}_{1}^{\ast }$ to ${E}_{2}^{\ast }$ at *τ* ≈ 0.77 (see blue curves); the stable states of subsystem (II) from initial frequencies of alleles (*p*_*I*,1_, *q*_*I*,1_, *p*_*U*,1_, *q*_*U*,1_) = (0.05, 0.05, 0.4, 0.5) switch from ${E}_{1}^{\ast }$ to ${E}_{4}^{\ast }$ at *τ* ≈ 0.93 (see red curves). Similarly, the bifurcation diagrams of the frequencies of alleles at equilibria of subsystem (II) from the three initial frequencies of alleles with respect to *ρ* are shown in Fig. 8. The stable states of subsystem (II) from some initial values may occur catastrophic change for resistance level *ρ* ∈ (0, 4*τ* − 3]. Based on the above bifurcation analyses, both parameters *τ* and *ρ* can significantly affect the switching behavior among the stable equilibria. Therefore, to further show how the two parameters *τ* and *ρ* affect the switching behavior, we take the two-parameter bifurcation analyses, as shown in Fig. S2. From which we can obtain the switching curves of stable equilibria from ${E}_{1}^{\ast }$ to ${E}_{2}^{\ast }$ (see from deep red region to green region) and from ${E}_{1}^{\ast }$ to ${E}_{4}^{\ast }$ (see from deep red to blue region). Note that the switching curves will alter with the change of initial frequencies of alleles, by comparing Fig. S2(a) with Fig. S2(b). By the same way, the *τ* − *ρ* plane can be drawn to obtain the switching curves of stable equilibria, corresponding to Figs 5, 6, 7 and 8, so here we omit it. Therefore, for any initial frequencies of alleles in mosquito populations, we can numerically obtain the corresponding switching curves of stable equilibria of subsystem (II), which is important for the control of mosquito populations.

### Replacement level and the density of uninfected mosquitoes

In the above subsections, the dynamic behaviors of subsystem (II) have been investigated by theoretical and numerical methods. Moreover, the two variables of subsystem (I) dependent upon the four variables of subsystem (II). In fact, in order to fight against dengue disease, we aim to realize the strategy of population replacement with high level and high speed. So in the following, we will focus on studying the effects of subsystem (II) and different parameter values on the dynamic behavior of subsystem (I). The detailed analysis of the existence and stabilities of equilibria for subsystem (I) is given in SI. And the expressions and stabilities of equilibria for subsystem (I) are shown in Table 6. Especially, the stabilities of ${\bar{E}}_{1}^{\ast }$ and ${\bar{E}}_{6}^{\ast }$ imply the success of complete population replacement or the failure of population replacement, respectively, which is determined by initial frequencies of alleles.

**Table 6:** The expressions and stabilities of equilibria for subsystem (I).

Equilibrium	${\bar{{\boldsymbol{E}}}}_{{\bf{0}}}^{{\boldsymbol{\ast }}}$	${\bar{{\boldsymbol{E}}}}_{{\bf{1}}}^{{\boldsymbol{\ast }}}$	${\bar{{\boldsymbol{E}}}}_{{\bf{0}}}^{({\bf{1}})}$	${\bar{{\boldsymbol{E}}}}_{{\bf{1}}}^{({\bf{2}})}$	${\bar{{\boldsymbol{E}}}}_{{\bf{2}}}^{{\boldsymbol{\ast }}}$	${\bar{{\boldsymbol{E}}}}_{{\bf{3}}}^{{\boldsymbol{\ast }}}$	${\bar{{\boldsymbol{E}}}}_{{\bf{4}}}^{{\boldsymbol{\ast }}}$	${\bar{{\boldsymbol{E}}}}_{{\bf{5}}}^{{\boldsymbol{\ast }}}$	${\bar{{\boldsymbol{E}}}}_{{\bf{6}}}^{{\boldsymbol{\ast }}}$
${I}_{i}^{\ast }$	0	0	0	0	$\tfrac{b\tau (1+\sqrt{{{\rm{\Delta }}}_{1}})}{2\delta T}$	$\tfrac{b\tau (1-\sqrt{{{\rm{\Delta }}}_{1}})}{2\delta T}$	$\tfrac{b\tau (\mathrm{(1}-\rho )+\sqrt{{{\rm{\Delta }}}_{2}})}{2\delta T\mathrm{(1}-\rho )}$	$\tfrac{b\tau (\mathrm{(1}-\rho )-\sqrt{{{\rm{\Delta }}}_{2}})}{2\delta T\mathrm{(1}-\rho )}$	$\tfrac{b}{\delta T}$
${U}_{i}^{\ast }$	0	$\tfrac{b}{\delta T}$	$\tfrac{b\mathrm{(1}-z)}{\delta T}$	$\tfrac{b}{\delta T}$	$\tfrac{b\tau (1-\sqrt{{{\rm{\Delta }}}_{1}})}{2\delta T}$	$\tfrac{b\tau (1+\sqrt{{{\rm{\Delta }}}_{1}})}{2\delta T}$	$\tfrac{b\tau (\mathrm{(1}-\rho )-\sqrt{{{\rm{\Delta }}}_{2}})}{2\delta T\mathrm{(1}-\rho )}$	$\tfrac{b\tau (\mathrm{(1}-\rho )+\sqrt{{{\rm{\Delta }}}_{2}})}{2\delta T\mathrm{(1}-\rho )}$	0
${\xi }_{i}^{\ast }$		0	0	0	$\tfrac{1+\sqrt{{{\rm{\Delta }}}_{1}}}{2}$	$\tfrac{1-\sqrt{{{\rm{\Delta }}}_{1}}}{2}$	$\tfrac{\mathrm{(1}-\rho )+\sqrt{{{\rm{\Delta }}}_{2}}}{\mathrm{2(1}-\rho )}$	$\tfrac{\mathrm{(1}-\rho )-\sqrt{{{\rm{\Delta }}}_{2}}}{\mathrm{2(1}-\rho )}$	1
Stability	S	S	U	S	S	U	S	U	S

S: Stable, U: Unstable, Δ_1_ = 1 − 4(1 − *τ*) ≥ 0, *i*.*e*. $\tau \ge \tfrac{3}{4}$ and Δ_2_ = (1 −* ρ*)^2^ − 4(1 −* ρ*) (1 −* τ*) ≥ 0, *i*.*e*. $\tau \ge \tfrac{3+\rho }{4}$.

In practice, *Wolbachia* infected mosquitoes should be released to realize the strategy of population suppression or population replacement. Based on Table 6, on the one hand, equilibrium ${\bar{E}}_{0}^{\ast }$ is locally stable, but not asymptotically stable. Thus, the success of population suppression occurs provided only the initial frequencies of alleles in the neighbourhood of ${E}_{0}^{\ast }$, which indicates that the probability of the failure of population suppression may be high. On the other hand, ${\bar{E}}_{1}^{\ast }$ and ${\bar{E}}_{i}^{\ast }(i=2,\,\mathrm{4)}$ coexist and are locally stable provided Δ_2_ > 0, which respectively means either the failure of population replacement or the success of partial population replacement determined by initial frequencies of alleles. Therefore, we aim to realize partial population replacement with high level, meanwhile, to keep the density of uninfected mosquitoes as low as possible. Particularly, the complete population replacement may be succeeded, provided perfect transmission rate (*τ* = 1).

Denote *ξ* = *I*/(*I* + *U*) to exactly depict replacement level. Then corresponding replacement levels of equilibria ${\bar{E}}_{i}^{\ast }(i=0,\,1,\,2,\,\ldots ,\,\mathrm{6)}$ are shown in Table 6. Here we just focus on the replacement levels of stable equilibria ${\bar{E}}_{i}^{\ast }(i=2,\,\mathrm{4)}$. Note that inequalities

$$\frac{1+\sqrt{{{\rm{\Delta }}}_{1}}}{2} > \frac{\mathrm{(1}-\rho )+\sqrt{{{\rm{\Delta }}}_{2}}}{\mathrm{2(1}-\rho )},\,\frac{b\tau (1-\sqrt{{{\rm{\Delta }}}_{1}})}{2\delta T} < \frac{b\tau (\mathrm{(1}-\rho )-\sqrt{{{\rm{\Delta }}}_{2}})}{2\delta T\mathrm{(1}-\rho )}$$

hold, so replacement level ${\xi }_{2}^{\ast }$ of ${\bar{E}}_{2}^{\ast }$ is higher than the level ${\xi }_{4}^{\ast }$ of ${\bar{E}}_{4}^{\ast },$ and the density of uninfected mosquitoes of ${\bar{E}}_{2}^{\ast }$ is less than that of ${\bar{E}}_{4}^{\ast }$. For convenience, replacement levels ${\xi }_{2}^{\ast }$ and ${\xi }_{4}^{\ast }$ are defined as high level with sensitive allele and low level with resistance allele, respectively.

**Figure 9:**
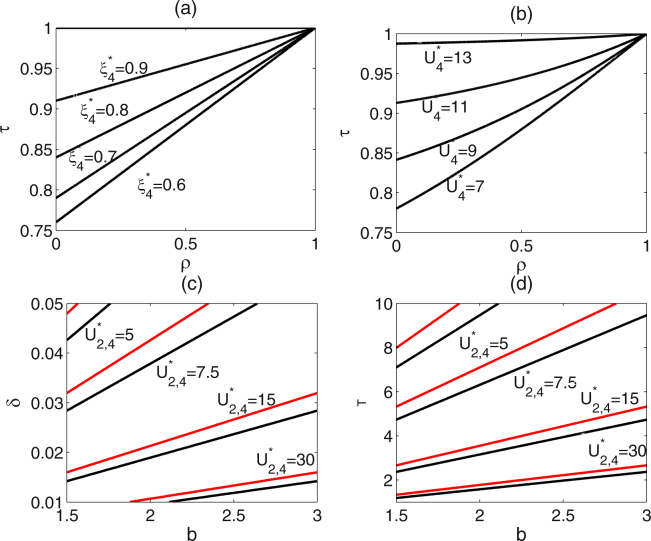
Contour plots of low replacement level with resistance allele and the densities of uninfected mosquitoes with respect to *Wolbachia*-induced parameters or demographic parameters. (**a**,**b**) Contour plots of ${\xi }_{4}^{\ast }$ and ${U}_{4}^{\ast }$ with respect to parameters *τ* and *ρ*. (**c**,**d**) Contour plots of both ${U}_{2}^{\ast }$ (see red curves) and ${U}_{4}^{\ast }$ (see black curves) with respect to parameters *b*,*δ* (**c**) and parameters *b*, *T* (**d**). Baseline parameter values are the same as those in Fig. 4.

In order to discuss the effect of each parameter on replacement level and the density of uninfected mosquitoes, all the parameters in system (2.3) are divided into two distinct types, one is *Wolbachia*-induced parameters, including *τ*, *ρ*, *h*, *z*, another is demographic parameters, including *b*, *δ*, *T*. Note that no matter subsystem (I) with or without fertility cost, the values of equilibria is not affected by parameters *z* and *h* except equilibrium ${\bar{E}}_{1}^{\mathrm{(1)}}$. Thus we do not consider the effects of the two parameters on the level of population replacement. According to the expressions of replacement levels ${\xi }_{2}^{\ast }$ and ${\xi }_{4}^{\ast }$, they are only affected by *Wolbachia*-induced parameter *τ* and the combination of *τ*, *ρ*, respectively. While the densities of uninfected mosquitoes ${U}_{2}^{\ast }$ and ${U}_{4}^{\ast }$ are affected by both *Wolbachia*-induced parameters and demographic parameters. High replacement level ${\xi }_{2}^{\ast }$ is increased with the increase of transmission rate. So the two parameter values are chosen and others are fixed, then we draw the contour plots of low replacement level ${\xi }_{4}^{\ast }$ and the densities of uninfected mosquitoes ${U}_{2}^{\ast }$ and ${U}_{4}^{\ast }$, as shown in Fig. 9.

It follows from Fig. 9(a) that low replacement level ${\xi }_{4}^{\ast }$ is increased with the increase of transmission rate *τ* and the decrease of resistance level *ρ*. Which indicates that high transmission rate and low resistance level contribute to improve low replacement level. Figure 9(b) shows that the density of uninfected mosquitoes is reduced with the decrease of transmission rate *τ* and the increase of resistance level *ρ*, which indicates that low transmission rate and high resistance level contribute to reduce the density of uninfected mosquitoes. So transmission rate and resistance level have reverse effects on replacement level and the density of uninfected mosquitoes. Therefore, in practice, suitable *Wolbachia* strains should be selected to balance the reverse effects so that population replacement can be realized with a relative high level and low density of uninfected mosquitoes. Figure 9(c,d) show that the densities of uninfected mosquitoes ${U}_{2}^{\ast }$ (see red lines) and ${U}_{4}^{\ast }$ (see black lines) are reduced with the decrease of birth rate *b* and the increase of death rate *δ* or reproductive period *T*.

### Replacement speed

**Figure 10:**
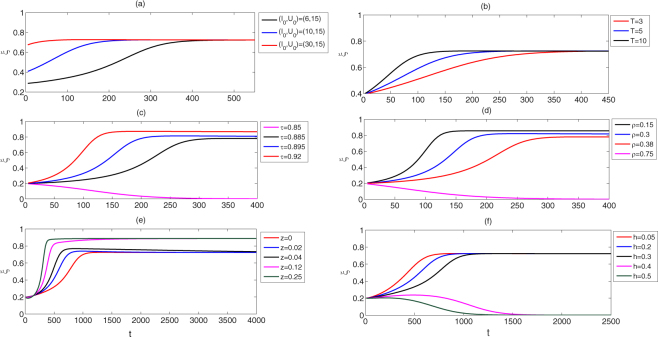
(**a**,**b**) The effects of different initial densities of infected mosquitoes and reproductive period on the speed of population replacement; (**c**,**d**) the effects of different transmission rates and resistance levels on the speed of population replacement; (**e**,**f**) the effects of different fertility cost and dominance levels on the speed of population replacement. Baseline parameter values are the same as those in Fig. 4.

In order to fight against dengue disease, it is important to study the effects of initial densities of mosquito populations and parameter values on the speed of population replacement. Therefore we numerically obtain the effects of these factors on the speed of the solutions of subsystem (I) stabilizing at stable states, as shown in Fig. 10. It follows from Fig. 10(a), when the density of uninfected mosquitoes is assumed as a constant, then the solutions of subsystem (I) from the more initial infected mosquitoes can stabilize at stable equilibria with less time. Figure 10(b) reflects that short reproduction period *T* contributes to the high speed of population replacement. Similarly, Fig. 10(c,d) indicate that high transmission rate *τ* and low resistance level *ρ* contribute to the high speed of population replacement.

Although fertility cost *z* and dominance level *h* have no effect on the values and stabilities of equilibria for subsystem (II), the two parameters may affect replacement level and speed, as shown in Fig. 10. Figure 10(e), successively choose fertility costs as *z* = 0, 0.02, 0.04, 0.12, 0.25 and fix other parameters. For the first three values of fertility cost, replacement levels of subsystem (I) stabilize at ${\xi }_{4}^{\ast }$ (red, blue and black curves), while for the last two values of fertility costs, replacement levels of subsystem (I) stabilize at ${\xi }_{2}^{\ast }$ (magenta and green curves). Moreover, high fertility cost benefits to improving the speed of population replacement and the success of high level replacement with sensitive allele because high fertility cost benefits to the expression intensity of CI. Similarly, in Fig. 10(f), we successively choose dominance levels as *h* = 0.05, 0.2, 0.3, 0.4, 0.5, and fix other parameters. For the first three values of dominance levels, replacement levels of subsystem (I) stabilize at ${\xi }_{4}^{\ast }$ (red, blue and black curves), while for the last two values of dominance levels, replacement levels of subsystem (I) stabilize at ${\xi }_{1}^{\ast }$ (magenta and green curves), which indicates the failure of population replacement. Moreover, low dominance level benefits to improving the speed of population replacement.

## Discussion

In this paper, based on Mendelian inheritance, we propose a multiscale model to study the effects of resistance allele of CI mechanism on dynamic behaviors of mosquito populations so that the strategies of population replacement with high level and speed can be succeeded. To establish the model, we first study the frequencies of three genotypes, the fitnesses of three genotypes, the genotype frequencies of new born offsprings and the frequencies of two alleles step by step. Then we formulate a six-dimension discrete system by the stroboscopic map of the original system. Further, based on the relation between subsystems (I) and (II), subsystem (II) is investigated at first, including the existence and stabilities of equilibria, basins of attraction of equilibria and parameter bifurcation analyses of *Wolbachia*-induced parameters. Finally, subsystem (I) is investigated, including the existence and stabilities of equilibria, the effects of initial frequencies of alleles and parameter values on the level and the speed of population replacement.

In practice, in order to control the spread of dengue virus, the strategy of population replacement should be succeeded at first. On the one hand, note that different parameter spaces of *τ* and *ρ* may lead to different cases for the coexistence of attractors. Therefore, based on special mosquito species, suitable *Wolbachia* strains need to be selected to ensure *Wolbachia*-induced parameters *τ* and *ρ* lying in the best region Ω_2_ or the second region Ω_3_, so that high level of population replacement may be realized (see Fig. 3). However, suppose *Wolbachia* strain fixed, there is a low probability for the success of high level replacement with sensitive allele (see deep green attractive region of ${E}_{2}^{\ast }$ in Fig. 4). In this case, *Wolbachia* strain should be reselected so that parameters *τ* and *ρ* lying in region Ω_3_, then corresponding mosquito control strategies should be carried out to drive initial frequencies of alleles in mosquito population lying in deep blue attractive region of ${E}_{4}^{\ast }$ in Fig. 4(b), which indicates the success of low level replacement with resistance allele. Especially, if some *Wolbachia* can induce perfect transmission rate, then the success of complete population replacement or the failure of population replacement is determined by the initial frequencies of alleles. Similarly, corresponding mosquito strategies should be carried out to drive initial frequencies of alleles in mosquito population lying in the objective basins of attraction for the success of complete population replacement. On the other hand, the switching curves for the stable equilibria of subsystem (II) are determined by initial frequencies of alleles. Therefore, suppose mosquito population confirmed, that means the initial density of mosquito population and initial frequencies of alleles in mosquitoes are fixed. Then based on special mosquito species, suitable *Wolbachia* strains should be selected to ensure *Wolbachia*-induced parameters *τ* and *ρ* lying in deep green regions (or second in deep blue regions in Fig. S2, so that high level replacement with sensitive allele (or low level replacement with resistance allele) can be realized.

Further, we aim to realize high level of population replacement with high speed and low density of uninfected mosquitoes which are determined by *Wolbachia*-induced parameters, demographic parameters of mosquitoes or initial frequencies of alleles in mosquitoes. According to Fig. 9, high transmission rate *τ* and low resistance level *ρ* contribute to improve the level of population replacement, while low transmission rate *τ*, high resistance level *ρ*, low birth rate *b*, high death rate *δ* and long reproduction period *T* benefit to reducing the density of uninfected mosquitoes. It follows from Fig. 10(a–d), high initial density of infected mosquitoes *I*_0_, high transmission rate *τ*, low resistance level *ρ* and low reproduction period *T* benefit to improving the speed of population replacement. It is worth noting that we should balance the inverse effects of parameters *τ*,*ρ* and *T* on control objectives. Especially, although fertility cost and dominance level has no effect on the level of replacement, the high fertility cost and low dominance level contribute to the success of population replacement with high speed.

In most empirical and experimental works, the evolution of CI is estimated by the intensity of CI expression^24,28,36^. Most researchers try to study the effects of some phenotypic factors on the evolution of CI and the distribution of host population. In addition, most mathematical models about CI expression focus on its macroscopic effects on the dynamics of host population^18,19,25,26,33^. Weeks *et al*.^37^ said that many factors can influence the expression of *Wolbachia* effects which is rarely considered in laboratory studies, and it is necessary to find a comprehensive approach to understand the host evolution. Thus, our hypothesis as well as multiscale model combine the host population and the microgenetic inheritance, which should be reasonable by theoretical support and actual possibility.

Note that the evolution of CI can greatly affect the spread and distribution of *Wolbachia* in some host populations. In this work, as far as we know, it is the first time to try exploring the relationship between mosquito population dynamics and CI genetic evolutionary dynamics. We assume that the introduction of *Wolbachia* infected mosquitoes in practice may lead to the evolution of mosquito population to resist *Wolbachia*-induced CI mechanism, which could affect the spread of *Wolbachia* in mosquitoes by the genetic development of the alleles related with CI mechanism. As it is the first step, we only focus on describing the intensity of CI expression by natural birth rate *b*, maternal transmission rate *τ* and the genetics parameters (here *z*, *h* and *ρ*) in different genotypes and different groups. In fact, the effects of the phenotypic factors on CI expression can be depicted by changing the values of these parameters in some extent.

This study shows that the evolution may weaken the ability of *Wolbachia* spread in mosquito popuation, and give some interpretation for the incomplete CI by genetic evolution, which may lead to the failure of *Wolbachia*-based control in some field trials. Consequently, the CI evolution in mosquito population should be aroused sustained attention. Tortosa *et al*.^38^ showed that incompatible matings effectively lower the fertility of infected males, so that selection acting on the host genome should tend to reduce the expression of CI in males, for example, by reducing the density of *Wolbachia* infection in males before sexual maturation. Thus, in the further work, we will explore the evolution of mosquito population with sex structure and some phenotypic factors to resist *Wolbachia*-induced CI mechanism, and its effect on the the spread of *Wolbachia* in mosquito populations. In addition, we will enhance the cooperation with the department of disease control to collect relative data of mosquito population with *Wolbachia* infection, and explore and develop the model to control other vector-borne diseases.

## Electronic supplementary material

Supplementary Information
