# Supplementary material for: Multiscale modelling the effects of CI genetic evolution in mosquito population on the control of dengue fever
Source: Sci Rep. 2017 Oct 24;7:13895. doi: 10.1038/s41598-017-13896-x (PMC5655163; doi:10.1038/s41598-017-13896-x)
Supplement: Supplementary file 1 — Supplementary Information [file 41598_2017_13896_MOESM1_ESM.pdf]

Supplementary Information (SI): Multiscale modelling the effects of CI genetic evolution in mosquito population on the control of dengue fever

Sha He♮ Xianghong Zhang♮ Juhua Liang♮ Sanyi Tang♮ [1]

♮ School of Mathematics and Information Science, Shaanxi Normal University, Xi'an, 710119, P.R. China

Content:

Supplementary Material and Methods

Supplementary Table S1

Supplementary Figures S1-S3

[1]Corresponding author. E-mail: sytang@snnu.edu.cn & sanyitang219@hotmail.com
Tel: +86(0)2985310232

1 Supplementary Material and Methods

1.1 Equilibria of subsystem (II)

The equilibria of subsystem (II) are determined by the following equations

$$\begin{cases} f_3(p_I, q_I, p_U, q_U) = p_I, \\ f_4(p_I, q_I, p_U, q_U) = q_I, \\ f_5(p_I, q_I, p_U, q_U) = p_U, \\ f_6(p_I, q_I, p_U, q_U) = q_U. \end{cases} \tag{S1}$$

Note that the four variables p_i and q_i $(i = I, U)$ being equal to zero satisfies equations (S1). It indicates that trivial equilibrium $E_0^* = (0, 0, 0, 0)$ always exists. The other equilibria of subsystem (II) can not be obtained directly owing to the complex form of (S1). Hence suppose that there is no fertility cost (*i.e.* $z = 0$), we obtain five boundary equilibria $E_i^*(i = 1, 2, \cdots, 5)$ as follows by solving equations (S1) under this special case.

$$\begin{cases} E_1^* = (0, 0, p_U, 1 - p_U), \\ E_2^* = \left(0, \frac{1+\sqrt{\Delta_1}}{2}, 0, \frac{1-\sqrt{\Delta_1}}{2}\right), E_3^* = \left(0, \frac{1-\sqrt{\Delta_1}}{2}, 0, \frac{1+\sqrt{\Delta_1}}{2}\right), \\ E_4^* = \left(\frac{(1-\rho)+\sqrt{\Delta_2}}{2(1-\rho)}, 0, \frac{(1-\rho)-\sqrt{\Delta_2}}{2(1-\rho)}, 0\right), E_5^* = \left(\frac{(1-\rho)-\sqrt{\Delta_2}}{2(1-\rho)}, 0, \frac{(1-\rho)+\sqrt{\Delta_2}}{2(1-\rho)}, 0\right), \end{cases} \tag{S2}$$

with $\Delta_1 = 1 - 4(1 - \tau) \geq 0$, *i.e.* $\tau \geq \frac{3}{4}$ and $\Delta_2 = (1 - \rho)^2 - 4(1 - \rho)(1 - \tau) \geq 0$, *i.e.* $\tau \geq \frac{3+\rho}{4}$. So transmission rate $\tau = 0.75$ is a necessary condition for the existence of above boundary equilibria of subsystem (II). Then we have the following main results.

Theorem 1.1 *For subsystem (II) with imperfect transmission rate* $\tau \in (0, 1)$ *and without fertility cost* (*i.e.* $z = 0$), *there exists one equilibrium cluster* E_1^* *and at most five equilibria* $E_i^*(i = 0, 2, \cdots, 5)$, *where* E_0^* *and* E_1^* *always exist. When* $\Delta_2 > 0$, *then* $E_i^*(i = 2, 3, 4, 5)$ *coexist; when* $\Delta_2 = 0$, *then* $E_i^*(i = 2, 3)$ *coexist,* E_4^* *and* E_5^* *collide together as* $(1/2, 0, 1/2, 0)$; *when* $\Delta_1 > 0$ *and* $\Delta_2 < 0$, *then* $E_i^*(i = 2, 3)$ *coexist,* E_4^* *and* E_5^* *disappear; when* $\Delta_1 = 0$, *then* E_1^* *and* E_2^* *collide together as* $(0, 1/2, 0, 1/2)$, E_4^* *and* E_5^* *disappear; when* $\Delta_1 < 0$, *then* $E_i^*(i = 2, 3, 4, 5)$ *disappear.*

Theorem 1.2 *For subsystem (II) with perfect transmission rate* $\tau = 1$ *and without fertility cost* (*i.e.* $z = 0$), *except equilibrium cluster* E_1^*, *there exists anther equilibrium cluster, denoted as* $E_6^* = (p_I, 1 - p_I, 0, 0)$.

In fact, in above special case $\tau = 1$, equilibria $E_i^*(i = 2, 3, 4, 5)$ are included in either E_1^* or E_6^*.

Next we investigate the existence of equilibria of subsystem (II) with imperfect transmission rate $\tau \in (0, 1)$ and fertility cost $z \in (0, 1]$. Based on $p_I + q_I + p_U + q_U = 1$, the roots of (S1) are determined by the first three equations, which can be rewritten as follows

$$\begin{pmatrix} f_{11} & f_{12} & f_{13} \\ f_{21} & f_{22} & f_{23} \\ f_{31} & f_{32} & f_{33} \end{pmatrix} \begin{pmatrix} a_1 \\ a_2 \\ a_3 \end{pmatrix} = \begin{pmatrix} 0 \\ 0 \\ 0 \end{pmatrix} \tag{S3}$$

with $a_1 = 1 - z,\ a_2 = (1 - hz),\ a_3 = 1$, and

$$\begin{aligned}
f_{11} &= p_{I,n}^3 + p_{I,n}p_{U,n}^2 + (1+\rho)p_{I,n}^2 p_{U,n} - \tau(p_{I,n}^2 + p_{I,n}p_{U,n}),\\
f_{12} &= 2p_{I,n}^2 q_{I,n} + p_{I,n}^2 q_{U,n} + p_{I,n}p_{U,n}q_{I,n} + 2p_{I,n}p_{U,n}q_{U,n} + \rho h(p_{I,n}p_{U,n}q_{I,n} + p_{I,n}^2 q_{U,n})\\
&\quad -\tau(p_{I,n}q_{I,n} + \tfrac{1}{2}p_{I,n}q_{U,n} + \tfrac{1}{2}p_{U,n}q_{I,n}),\\
f_{13} &= p_{I,n}q_{I,n}^2 + p_{I,n}q_{I,n}q_{U,n} + p_{I,n}q_{U,n}^2,\\
f_{21} &= p_{I,n}^2 q_{I,n} + q_{I,n}p_{U,n}^2 + (1+\rho)p_{I,n}q_{I,n}p_{U,n},\\
f_{22} &= 2p_{I,n}q_{I,n}^2 + p_{I,n}q_{I,n}q_{U,n} + p_{U,n}q_{I,n}q_{I,n} + 2p_{I,n}p_{U,n}q_{U,n} + \rho h(p_{I,n}p_{U,n}q_{I,n} + p_{I,n}^2 q_{U,n})\\
&\quad -\tau(p_{I,n}q_{I,n} + \tfrac{1}{2}p_{I,n}q_{U,n} + \tfrac{1}{2}p_{U,n}q_{I,n}),\\
f_{23} &= q_{I,n}^3 + q_{I,n}^2 q_{U,n} + q_{I,n}q_{U,n}^2 - \tau(q_{I,n}^2 + q_{I,n}q_{U,n}),\\
f_{31} &= p_{I,n}^2 p_{U,n} + p_{U,n}^3 + (1+\rho)p_{I,n}p_{U,n}^2 - (1-\tau)(p_{I,n}^2 + p_{I,n}p_{U,n}) - p_{U,n}^2 - \rho p_{U,n}p_{I,n},\\
f_{32} &= 2p_{I,n}q_{I,n}p_{U,n} + p_{I,n}q_{U,n}p_{U,n} + p_{U,n}^2 q_{I,n} + 2p_{U,n}^2 q_{U,n} + \rho h(p_{U,n}^2 q_{I,n} + p_{I,n}q_{U,n}p_{U,n})\\
&\quad -(1-\tau)(p_{I,n}q_{I,n} + \tfrac{1}{2}p_{I,n}q_{U,n} + \tfrac{1}{2}p_{U,n}q_{I,n}) - p_{U,n}q_{U,n} - \tfrac{1}{2}\rho h(p_{U,n}q_{I,n} + p_{I,n}q_{U,n}),\\
f_{33} &= q_{I,n}^2 p_{U,n} + q_{I,n}q_{U,n}p_{U,n} + q_{U,n}^2 p_{U,n}.
\end{aligned} \tag{S4}$$

In the following, we first prove that the values of $a_i(i = 1, 2, 3)$ have no effect on the nonzero parts of equilibria $E_i^*(i = 2, 3)$ and $E_i^*(i = 4, 5)$ for subsystem (II) with imperfect transmission rate $\tau \in (0, 1)$ and without fertility cost (*i.e.* $z = 0$). In fact, if $z = 0$ holds, then $a_1 = a_2 = a_3 = 1$. Based on (S4), equilibria E_1^*, $E_i^*(i = 2, 3)$ and $E_i^*(i = 4, 5)$ satisfied the following equations (S5), (S6) and (S7), respectively.

$$f_{i,j} = 0\ (i = 1, 2; j = 1, 2, 3), f_{31} = p_U^3 - p_U^2, f_{32} = 2p_U^2 q_U - p_U q_U, f_{33} = p_U q_U^2. \tag{S5}$$

$$f_{i,j} = 0\ (i = 1, 3; j = 1, 2, 3), f_{21} = f_{22} = 0, f_{23} = q_I(q_I^2 - q_I + 1 - \tau). \tag{S6}$$

$$f_{i,j} = 0\ (i = 2,3; j = 1,2,3), f_{11} = p_I((1-\rho)p_I^2 - (1-\rho)p_I + 1 - \tau), f_{22} = f_{32} = 0. \quad \text{(S7)}$$

Hence nonzero parts of E_1^*, $E_i^*(i = 2,3)$ and $E_i^*(i = 4,5)$ are determined by $f_{31} + f_{32} + f_{33} = 0$, $f_{23} = 0$ and $f_{11} = 0$, respectively, which indicates that there is no effect for the values of a_i $(i = 1,2,3)$ on the nonzero parts of $E_i^*(i = 2,3)$ and $E_i^*(i = 4,5)$. That is to say, no matter that fertility cost exists or not, there exist the same equilibria $E_i^*(i = 2,3)$ and $E_i^*(i = 4,5)$. While equilibrium E_1^* of subsystem (II) with fertility cost is determined by the following equations

$$\begin{cases} a_1 f_{31} + a_2 f_{32} + a_3 f_{33} = p_U(p_U + q_U)(p_U + q_U - 1) \\ \qquad\qquad + z q_U((1-2h)q_U^2 + (3h-2)q_U + (1-h)) = 0, \\ p_U + q_U = 1. \end{cases} \quad \text{(S8)}$$

Solving above equations with respect to p_U and q_U, one yields two meaningful roots, denoted as $p_U^{(1)} = 1, q_U^{(1)} = 0$, and $p_U^{(2)} = 0, q_U^{(2)} = 1$. So equilibrium cluster E_1^* is degenerated into two equilibria for subsystem (II) with fertility cost $z \in (0,1]$, denoted as $E_1^{(1)} = (0,0,1,0)$ and $E_1^{(2)} = (0,0,0,1)$.

Therefore, we have the following main results.

Theorem 1.3 *For subsystem (II) with imperfect transmission rate* $\tau \in (0,1)$ *and fertility cost* $z \in (0,1]$, *there are at most seven equilibria* $E_1^{(j)}(j = 1,2)$ *and* $E_i^*(i = 0,2,3,4,5)$, *where* $E_1^{(1)} = (0,0,1,0)$ *and* $E_1^{(2)} = (0,0,0,1)$, *while others are the same as those of without fertility cost.*

Similarly, according to (S3), for subsystem (II) with perfect transmission rate $\tau = 1$ and fertility cost $z \in (0,1]$, there exist three equilibria E_0^* and $E_1^{(j)}(j = 1,2)$ in this case. While compared with those of transmission rate $\tau \in (0,1)$ and fertility cost $z \in (0,1]$, others equilibria will disappear in this special case.

1.2 Stability of equilibria for subsystem (II)

In the neighborhood of equilibria, the dynamics of discrete subsystem (II) is determined by the linearization

$$X_{n+1} = JX_n,$$

with Jacobian matrix J as the linear counterpart of subsystem (II). For convenience, we denote the eigenvalues of equilibria E_i^* as $\lambda_{i,j}(i = 0, 1, 2\cdots, 6;\ j = 1, 2, 3, 4)$, respectively. Taking the derivative of $f_k(p_I, q_I, p_U, q_U)$ $(k = 3, 4, 5, 6)$ with respect to p_I, q_I, p_U, q_U, then we can obtain the Jacobian matrix of subsystem (II) as follows

$$J = \frac{1}{1+Q}E + \frac{1}{(1+Q)^2}\begin{pmatrix} g_1(p_I) & g_1(q_I) & g_1(p_U) & g_1(q_U) \\ g_2(p_I) & g_2(q_I) & g_2(p_U) & g_2(q_U) \\ g_3(p_I) & g_3(q_I) & g_3(p_U) & g_3(q_U) \\ g_4(p_I) & g_4(q_I) & g_4(p_U) & g_4(q_U) \end{pmatrix}, \tag{S9}$$

where E is a fourth-order unit matrix,

$$\begin{aligned} g_1(x) &= \tau\tfrac{\partial A}{\partial x}(1+Q) - (p_I + \tau A)\tfrac{\partial Q}{\partial x}, \\ g_2(x) &= \tau\tfrac{\partial B}{\partial x}(1+Q) - (q_I + \tau B)\tfrac{\partial Q}{\partial x}, \\ g_3(x) &= \left((1-\tau)\tfrac{\partial A}{\partial x} + \tfrac{\partial C}{\partial x}\right)(1+Q) - (p_U + (1-\tau)A + C)\,\tfrac{\partial Q}{\partial x}, \\ g_4(x) &= \left((1-\tau)\tfrac{\partial B}{\partial x} + \tfrac{\partial D}{\partial x}\right)(1+Q) - (q_U + (1-\tau)B + D)\,\tfrac{\partial Q}{\partial x}, \end{aligned} \tag{S10}$$

with $x \in (p_I, q_I, p_U, q_U)$. By simple calculations, the eigenvalues of all the equilibria $E_i^*(i = 0, 1, 2\cdots, 6)$ are listed in Tab. S1.

Since all eigenvalues $\lambda_{0,j}(j = 1, 2, 3, 4)$ of equilibrium E_0^* are equal to one, E_0^* is locally stable, but not asymptotically stable. While $\lambda_{1,1} = 1$ and $|\lambda_{1,i}| < 1(i = 2, 3, 4)$ hold, then E_1^* is locally stable, but not asymptotically stable, so there exists a stable manifold for it. For equilibrium $E_1^{(1)}$, it is unstable because eigenvalue $\lambda_{1,2}^{(1)} = (1 + b - bhz)/(1 + b - bz)$ is larger than one when both parameters z, h lie in $(0, 1]$. For equilibrium $E_1^{(2)}$, it is asymptotically stable due to all eigenvalues being less than one when all parameters τ, z, h lie in $(0, 1]$.

When transmission rate is perfect, *i.e.* $\tau = 1$, it is easy to prove that inequalities $0 < |\lambda_{6,4}| \le |\lambda_{6,3}|$ hold. Thus, the stability of E_6^* is determined by the relation between $\lambda_{6,3}$ and one. To compare $\lambda_{6,3}$ with one, assuming that $\lambda_{6,3} \le 1$, *i.e.* $(M_5 + N_5) \le (1 + b)$ holds, then we have a series of identical deformations as follows

$$\begin{aligned} &(M_5 + N_5) \le (1 + b) \\ \Longleftrightarrow\ & \tfrac{1}{4}h\rho + \tfrac{1}{2}\rho p_I + \tfrac{1}{4}\sqrt{-8h\rho^2 p_I^2 + h^2\rho^2 + 4h\rho^2 p_I + 4\rho^2 p_I^2} \le 1, \\ \Longleftrightarrow\ & \tfrac{1}{16}(-8h\rho^2 p_I^2 + h^2\rho^2 + 4h\rho^2 p_I + 4\rho^2 p_I^2) \le (1 - \tfrac{1}{4}h\rho - \tfrac{1}{2}\rho p_I)^2, \\ \Longleftrightarrow\ & -\tfrac{1}{2}h\rho^2 p_I^2 + \tfrac{1}{2}h\rho + \rho p_I - 1 \le 0. \end{aligned} \tag{S11}$$

In order to prove that inequality $-\frac{1}{2}h\rho^2 p_I^2 + \frac{1}{2}h\rho + \rho p_I - 1 \leq 0$ always holds, we denote $f(h,\rho,p_I) = -\frac{1}{2}h\rho^2 p_I^2 + \frac{1}{2}h\rho + \rho p_I - 1$. According to extreme value theorem, it is easy confirm that $f(h,\rho,p_I)$ reaches its maximum value 0 at point $(1,1,1)$. Note that parameters h and ρ lie in [0,1], so we have $f(h,\rho,p_I) \leq 0$, which indicates (S11) holds. Therefore, $|\lambda_{6,j}| \leq 1, (j = 3,4)$ always holds. Combination with $\lambda_{6,1} = 1$ and $|\lambda_{6,2}| < 1$, then E_6^* is locally stable, but not asymptotically stable.

While for the stabilities of other equilibria $E_i^*(i = 2,3,4,5)$, although the eigenvalues of Jacobian matrix at them can be calculated, it is difficult to judge the relation between the modulus of eigenvalues and one owing to their complex form. So we investigate the stabilities of equilibria $E_i^*(i = 2,3,4,5)$ by numerical method. Consider that the frequencies of alleles in infected and uninfected mosquitoes are less than or equal to one and the sum of them is equal to one, so the meaningful region of three variables seems a triangular pyramid, denoted as $\Omega = \{p_I \geq 0, q_I \geq 0, p_U \geq 0, q_U \geq 0, p_I + q_I + p_U + q_U \leq 1\}$. The solutions of subsystem (II) from different initial values may stabilize at different equilibria as shown in Fig. S1 and Fig. 5, which indicate the local stabilities of those equilibria.

Note that some results listed in the following remarks are only verified by numerical methods without rigorous mathematical justifications. Thus, all of these statements are expressed as the form of remarks instead of theorems.

Remark 1.1 *For subsystem (II) with imperfect transmission rate* $\tau \in (0,1)$ *and without fertility cost* ($i.e.$ $z = 0$), E_0^* *is locally stable, but not asymptotically stable.*
(1) If $\Delta_2 > 0$ ($i.e.$ Ω_3), *then* $E_i^*(i = 1,2,4)$ *are locally stable, while* E_3^* *and* E_5^* *are unstable;*
(2) If $\Delta_1 > 0, \Delta_2 < 0$ ($i.e.$ Ω_2), *then* $E_i^*(i = 1,2)$ *are locally stable, while* E_3^* *is unstable;*
(3) If $\Delta_1 < 0$ ($i.e.$ Ω_1), *then* E_1^* *is locally stable.*

Remark 1.2 *For subsystem (II) with perfect transmission rate* $\tau = 1$ *and without fertility cost* ($i.e.$ $z = 0$), E_0^* *and* E_6^* *are locally stable, but not asymptotically stable, equilibrium clusters* E_1^* *is locally stable.*

Remark 1.3 *For subsystem (II) with imperfect transmission rate* $\tau \in (0,1)$ *and fertility cost, i.e.* $z \in (0,1]$, $E_1^{(1)}$ *is unstable,* $E_1^{(2)}$ *is locally stable, while the stabilities of others are the same as those of without fertility cost.*

Similarly, the stabilities of equilibria for subsystem (II) with perfect transmission rate $\tau = 1$ and fertility cost $z \in (0,1]$ can be investigated.

1.3 The existence and stability of equilibrium of subsystem (I)

The equilibria of subsystem (I) are determined by the following equations

$$\begin{cases} f_1(I, U, p_I, q_I, p_U, q_U) = I, \\ f_2(I, U, p_I, q_I, p_U, q_U) = U, \end{cases} \tag{S12}$$

where the values of variables p_I, q_I, p_U and q_U are corresponded to those of equilibria $E_i^*(i = 0, 1, 2, \cdots, 6)$ for subsystem (II), and can be treated as four parameters in subsystem (I). For simplification, the terms Q_1 and Q_2 with respect to the four variables are also treated as parameters in the above equations. Solving equations (S12) with respect to I and U, we obtain equilibrium $\bar{E}^* = (I^*, U^*)$, with

$$I^* = \frac{\tau Q_1}{\delta T}, \; U^* = \frac{(1-\tau)Q_1 + Q_2}{\delta T}.$$

Substituting the values of equilibria $E_i^*(i = 0, 1, 2, \cdots, 5)$ into the expressions of I^* and U^* yields five corresponding equilibria $\bar{E}_i^*(i = 0, 1, 2, \cdots, 5)$, as shown in Tab. 6.

By simple calculation, the eigenvalues of Jacobian matrix of subsystem (I) at equilibrium $\bar{E}^*$ are denoted as $\bar{\lambda}_i$ $(i = 1, 2)$, with

$$0 < \bar{\lambda}_1 = \bar{\lambda}_2 = \frac{1}{Q_1 + Q_2 + 1} < 1.$$

Based on the relationship between subsystems (I) and (II), each equilibrium and its stability of subsystem (I) are corresponded to that of subsystem (II), which indicates that if the equilibrium of subsystem (II) is stable (unstable), then the corresponding equilibrium of subsystem (I) is also stable (unstable). Accordingly, the biological significance of stable equilibria $\bar{E}_i^*(i = 0, 1, 2, 4)$ are the extinction of mosquito population, the failure of population replacement, the partial replacement with success of sensitive allele and partial replacement with success of resistance allele, respectively.

Therefore, we have the following main results.

Remark 1.4 *For subsystem (I) with imperfect transmission rate* $\tau \in (0, 1)$ *and without fertility cost* ($i.e.$ $z = 0$), *there are at most six equilibria* $\bar{E}_i^*(i = 0, 1, 2, \cdots, 5)$, *where* $\bar{E}_0^*$ *is locally stable, but not asymptotically stable.*

(1) If $\Delta_2 > 0$ ($i.e.$ Ω_3), *then* $\bar{E}_i^*(i = 0, 1, 2, \cdots, 5)$ *coexist, where* $\bar{E}_i^*(i = 1, 2, 4)$ *are locally*

stable, while $\bar{E}_3^*$ *and* $\bar{E}_5^*$ *are unstable;*

(2) If $\Delta_1 > 0, \Delta_2 < 0$ $(i.e.\ \Omega_2)$, *then* $\bar{E}_i^*(i = 0, 1, 2, 3)$ *coexist, where* $\bar{E}_i^*(i = 1, 2)$ *are locally stable, while* $\bar{E}_3^*$ *is unstable;*

(3) If $\Delta_1 < 0$ $(i.e.\ \Omega_1)$, *then* $\bar{E}_i^*(i = 0, 1)$ *coexist, where* $\bar{E}_1^*$ *is locally stable.*

While for subsystem (I) with imperfect transmission rate $\tau \in (0, 1)$ *and fertility cost* $z \in (0, 1]$, *equilibrium cluster* $\bar{E}_1^*$ *degenerates to* $\bar{E}_1^{(1)}$ *and* $\bar{E}_1^{(2)}$, *where* $\bar{E}_1^{(1)}$ *is unstable, and* $\bar{E}_1^{(2)}$ *is locally stable. The existence and stability of others are same as those of without fertility cost.*

Based on Remark 1.4, there exists the coexistence of three attractors provided $\Delta_2 > 0$ $(i.e.\ \Omega_3)$. So Fig. S3 shows the effects of different initial frequencies of alleles and initial densities of mosquito population on the solutions of subsystem (I).

Remark 1.5 *For subsystem (I) with perfect transmission rate* $\tau = 1$ *and without fertility cost* $(i.e.\ z = 0)$, *there exist two stable equilibria* $\bar{E}_1^*$ *and* $\bar{E}_6^*$ *corresponding with those of subsystem (II).*

2 Supplementary Table S1

Table S1: Eigenvalues of Jacobian matrix J at equilibria of subsystem (II).

Eigenvalue	$\lambda_{i,1}$	$\lambda_{i,2}$	$\lambda_{i,3}$	$\lambda_{i,4}$
$J\|_{E_0^*}$	1	1	1	1
$J\|_{E_1^*}$	1	$\frac{1}{1+b}$	$\frac{1+b\tau}{1+b}$	$\frac{2+b\tau}{2(1+b)}$
$J\|_{E_1^{(1)}}$	$\frac{1}{1+b-bz}$	$\frac{1+b-bhz}{1+b-bz}$	$\frac{1+b\tau-bz\tau}{1+b-bz}$	$\frac{2+b\tau-bhz\tau}{2(1+b-bz)}$
$J\|_{E_1^{(2)}}$	$\frac{1}{1+b}$	$\frac{1+b-bhz}{1+b}$	$\frac{1+b\tau}{1+b}$	$\frac{2+b\tau-bhz\tau}{2(1+b)}$
$J\|_{E_2^*}$	$\frac{1}{1+b\tau}$	$\frac{3b+2-2b\tau-b\sqrt{\Delta_1}}{2(1+b\tau)}$	$\frac{M_1\sqrt{\Delta_1}+N_1+b\sqrt{M_2\sqrt{\Delta_1}+N_2}}{8(1+b\tau)}$	$\frac{M_1\sqrt{\Delta_1}+N_1-\sqrt{M_2\sqrt{\Delta_1}+N_2}}{8(1+b\tau)}$
$J\|_{E_3^*}$	$\frac{1}{1+b\tau}$	$\frac{3b+2-2b\tau+b\sqrt{\Delta_1}}{2(1+b\tau)}$	$\frac{-M_1\sqrt{\Delta_1}+N_1+b\sqrt{-M_2\sqrt{\Delta_1}+N_2}}{8(1+b\tau)}$	$\frac{-M_1\sqrt{\Delta_1}+N_1-\sqrt{-M_2\sqrt{\Delta_1}+N_2}}{8(1+b\tau)}$
$J\|_{E_4^*}$	$\frac{1}{1+b\tau}$	$\frac{3b+2-2b\tau+b\rho-b\sqrt{\Delta_2}}{2(1+b\tau)}$	$\frac{M_3\sqrt{\Delta_2}+N_3+b\sqrt{M_4\sqrt{\Delta_2}+N_4}}{8(1+b\tau)(\rho-1)}$	$\frac{M_3\sqrt{\Delta_2}+N_3-\sqrt{M_4\sqrt{\Delta_2}+N_4}}{8(1+b\tau)(\rho-1)}$
$J\|_{E_5^*}$	$\frac{1}{1+b\tau}$	$\frac{3b+2-2b\tau+b\rho+b\sqrt{\Delta_2}}{2(1+b\tau)}$	$\frac{-M_3\sqrt{\Delta_2}+N_3+b\sqrt{-M_4\sqrt{\Delta_2}+N_4}}{8(1+b\tau)(\rho-1)}$	$\frac{-M_3\sqrt{\Delta_2}+N_3-\sqrt{-M_4\sqrt{\Delta_2}+N_4}}{8(1+b\tau)(\rho-1)}$
$J\|_{E_6^*}$	1	$\frac{1}{1+b}$	$\frac{M_5+N_5}{1+b}$	$\frac{M_5-N_5}{1+b}$

with $M_1 = b(h\rho - 1)$, $N_1 = bh\rho + 2b\tau + 3b + 8$,
$M_2 = 2h^2\rho^2 - 12h\rho\tau + 4h\rho + 12\tau - 6$,
$N_2 = 4h^2\rho^2\tau - 2h^2\rho^2 - 32h\rho\tau^2 + 12h\rho\tau + 12h\rho + 36\tau^2 - 32\tau + 6$,
$M_3 = b(1 - h\rho)$, $N_3 = bh\rho^2 - bh\rho + 2b\tau\rho + 3b\rho - 2b\tau - 3b + 8\rho - 8$,
$M_4 = -2h^2\rho^3 + 2h^2\rho^2 - 4h\rho^2 + 12h\rho^2\tau - 12h\rho\tau + 4h\rho - 12\rho\tau + 6\rho + 12\tau - 6$,
$N_4 = -4h^2\rho^3\tau + 4h^2\rho^2\tau - 12h\rho^3\tau + 32h\rho^2\tau^2 - 32h\rho\tau^2 + 12h\rho\tau + 2h^2\rho^4 - 2h^2\rho^2 + 4h\rho^3 + 4\rho^2\tau^2 - 16h\rho^2 - 4\rho^2\tau - 40\rho\tau^2 + 12h\rho + 10\rho^2 + 36\rho\tau + 36\tau^2 - 16\rho - 32\tau + 6$,
$M_5 = \frac{1}{4}bh\rho + \frac{1}{2}b\rho p_I + 1$, $N_5 = \frac{b}{4}\sqrt{-8h\rho^2 p_I^2 + h^2\rho^2 + 4h\rho^2 p_I + 4\rho^2 p_I^2}$.

3 Supplementary Figures S1-S3

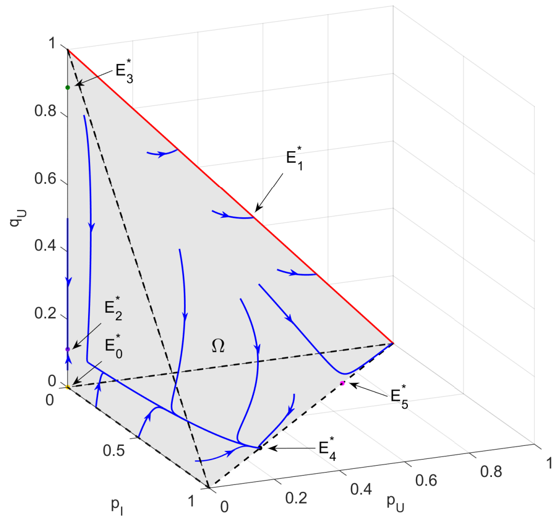

Figure S1:The effects of initial frequencies of alleles on the solutions of subsystem (II). Gray region Ω is meaningful for subsystem (II). Parameter values are fixed as follows: $b = 2, \delta = 0.03, T = 5, \tau = 0.9, \rho = 0.5, h = 0.35, z = 0$. $\Omega = \{p_I \geq 0, q_I \geq 0, p_U \geq 0, q_U \geq 0, p_I + q_I + p_U + q_U \leq 1\}$.

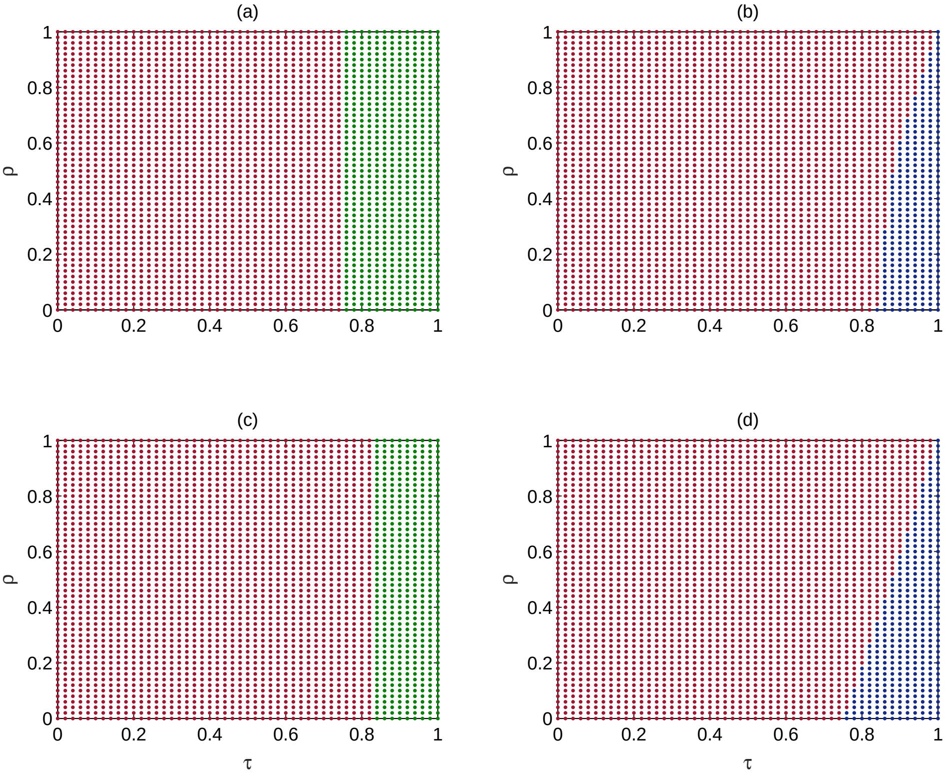

Figure S2: The catastrophic shifts from stable equilibria E_1^* to E_2^* (A-C), and from E_1^* to E_4^* (B-D) for subsystem (II) corresponding in Fig. 6 and Fig. 8. When parameter values are chosen from the deep red, green and blue regions, then the solutions of subsystem (II) stabilize at equilibria E_1^*, E_2^* and E_4^*, respectively. Baseline parameter values are the same as those in Fig. 6.

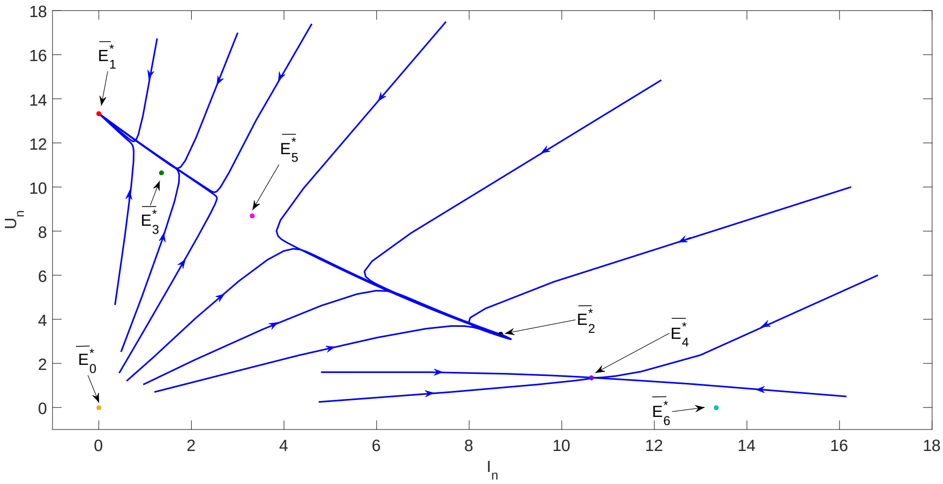

Figure S3: The effects of different initial frequencies of alleles and densities of mosquitoes on the solutions of subsystem (I). Parameter values are the same as those in Fig. 4.
